# Function and evolution of local repeats in the *Firre* locus

**DOI:** 10.1038/ncomms11021

**Published:** 2016-03-24

**Authors:** Ezgi Hacisuleyman, Chinmay J. Shukla, Catherine L. Weiner, John L. Rinn

**Affiliations:** 1 Department of Molecular and Cellular Biology, Harvard University, Cambridge, Massachusetts 02138, USA; 2 Department of Stem Cell and Regenerative Biology, Harvard University7 Divinity Avenue, Room 305, Cambridge, Massachusetts 02138, USA; 3 Broad Institute of Massachusetts Institute of Technology and Harvard, Cambridge, Massachusetts 02142, USA; 4 Department of Biological and Biomedical Sciences, Harvard University, Boston, Massachusetts 02115, USA; 5 Department of Pathology, Beth Israel Deaconess Medical Center, Boston, Massachusetts 02215, USA

## Abstract

More than half the human and mouse genomes are comprised of repetitive sequences, such as transposable elements (TEs), which have been implicated in many biological processes. In contrast, much less is known about other repeats, such as local repeats that occur in multiple instances within a given locus in the genome but not elsewhere. Here, we systematically characterize local repeats in the genomic locus of the Firre long noncoding RNA (lncRNA). We find a conserved function for the RRD repeat as a ribonucleic nuclear retention signal that is sufficient to retain an otherwise cytoplasmic mRNA in the nucleus. We also identified a repeat, termed R0, that can function as a DNA enhancer element within the intronic sequences of Firre. Collectively, our data suggest that local repeats can have diverse functionalities and molecular modalities in the *Firre* locus and perhaps more globally in other lncRNAs.

## Abstract (web summary)

Mammalian genomes contain multiple repetitive sequences such as transposable elements and local repeats. Here, the authors show that the conserved long non-coding RNA Firre contains repeats that act as nuclear retention signals and a DNA enhancer element.

## Graphical abstract

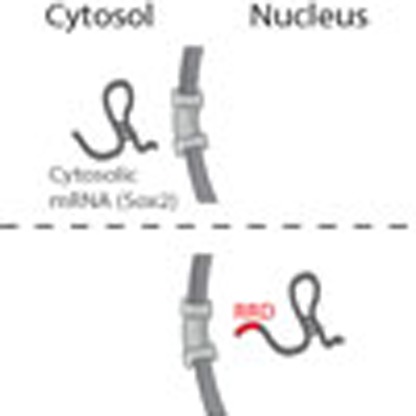

Most of our genome is comprised of repetitive elements, such as transposable elements (TEs), tandem repeats (TRs) and local repeats (LRs)^1^,^2^,^3^. Recent research has revealed that TEs can play important roles in transcriptional and post-transcriptional regulation^4^,^5^,^6^,^7^,^8^,^9^,^10^,^11^,^12^,^13^,^14^. In addition to TEs, TRs and LRs also encompass a large portion of the genome (for example, 14% of all protein-coding genes contain TRs)^15^,^16^. TRs are defined as sequences that repeat adjacent to each other or in a continuous manner. LRs are defined as sequences that recur multiple times in a discontinuous manner within a single locus and are not found elsewhere in the genome. LRs vary from 100 to 750 bp long occurring both in exons (exonic LRs), most likely functioning in the RNA transcript, and in introns (intronic LRs), potentially encoding DNA regulatory elements. Although TRs have well-established roles in the regulation of coding and noncoding (lncRNA) genes^7^,^17^,^18^,^19^,^20^,^21^,^22^,^23^,^24^,^25^, LRs remain virtually unstudied in this regard^26^. Recent studies have identified LRs on the X chromosome with roles in epigenetic and three-dimensional organization regulation^27^,^28^,^29^,^30^,^31^,^32^,^33^,^34^,^35^,^36^,^37^. For example, the *DXZ4*, *X56* and *FIRRE* loci on the X chromosome comprise numerous LRs^27^,^28^,^29^,^30^,^31^,^32^,^33^,^34^,^35^,^36^,^37^,^38^. Of these regions, *X56* and *FIRRE* produce lncRNAs^27^,^28^, are localized in three-dimensional proximity^39^ and exhibit distinctive epigenetic features^27^,^29^. *FIRRE* also exhibits allele-specific epigenetic states between males and females^27^,^28^,^29^,^40^,^41^. For instance, the architectural insulator protein, CCCTC-binding factor (CTCF), along with the transcription factor (TF) YY1, have been found to be associated with the *FIRRE* locus in an allele-specific manner^28^,^29^,^40^,^41^. Yet, despite these interesting features, the functional roles and characteristics of LRs remain unresolved.

We previously found that Firre, is required for pluripotency and adipogenesis^27^,^42^ and harbours many LRs, including one that comprises the majority of its exons^27^. Further underscoring the importance of this locus, the amplification of *FIRRE*, along with *IGSF1* and *OR13H1*, has been genetically implicated in a brain malformation, termed periventricular nodular heterotopia, in humans^43^.

Here, we further explore the molecular functionalities of LRs (both DNA and RNA forms) in the *Firre* locus across multiple species to gain deeper insights into the LR biology. We found that LRs, but not TRs, are enriched in lncRNA loci. Detailed experimental investigation revealed that the exonic LR, repeating RNA domain (RRD), is both necessary and sufficient as a ribonucleic nuclear retention signal. We further characterized a second repeat, R0, that recruits CTCF and RAD21. Moreover, we find that R0 can function as a DNA enhancer element. Collectively, our findings demonstrate the functional importance of LRs in the *Firre* locus that may extend to many additional LR-containing lncRNAs.

## Results

### LncRNAs are enriched in LRs

We first set out to characterize the LR and TR instances in mRNAs and lncRNAs (GENCODE v19 (ref. 44) and lncRNAs from a previously published catalogue^45^). TRs are repeated adjoining sequences that occur at multiple regions in the genome. LRs, on the other hand, are defined as sequences that repeat within one given genomic locus, in a non-continuous manner (for example, separated by non-repeating sequences), but not elsewhere in the genome. To examine the TR and LR contents in lncRNA and mRNA loci, we removed all TEs based on RepeatMasker as they are not LRs or TRs (see Methods for details). Then, we performed RepeatScout on the TE masked files to identify repetitive substrings in a given DNA sequence^46^. RepeatScout finds all instances of k-mers of specified length (16 in this case) and extends them in both directions to find repeating substrings in a given DNA sequence. To explicitly call TRs from the RepeatScout output, we used Tandem Repeat Finder (TRF)^47^. Separately, we used TRF to also find TRs in the TE-masked sequence for each gene. Next, we normalized for mRNA and lncRNA length (as mRNAs are significantly longer (Supplementary Fig. 1A)). Finally, we calculated the number of LRs and TRs present in each protein-coding and lncRNA gene.

**Figure 1:**
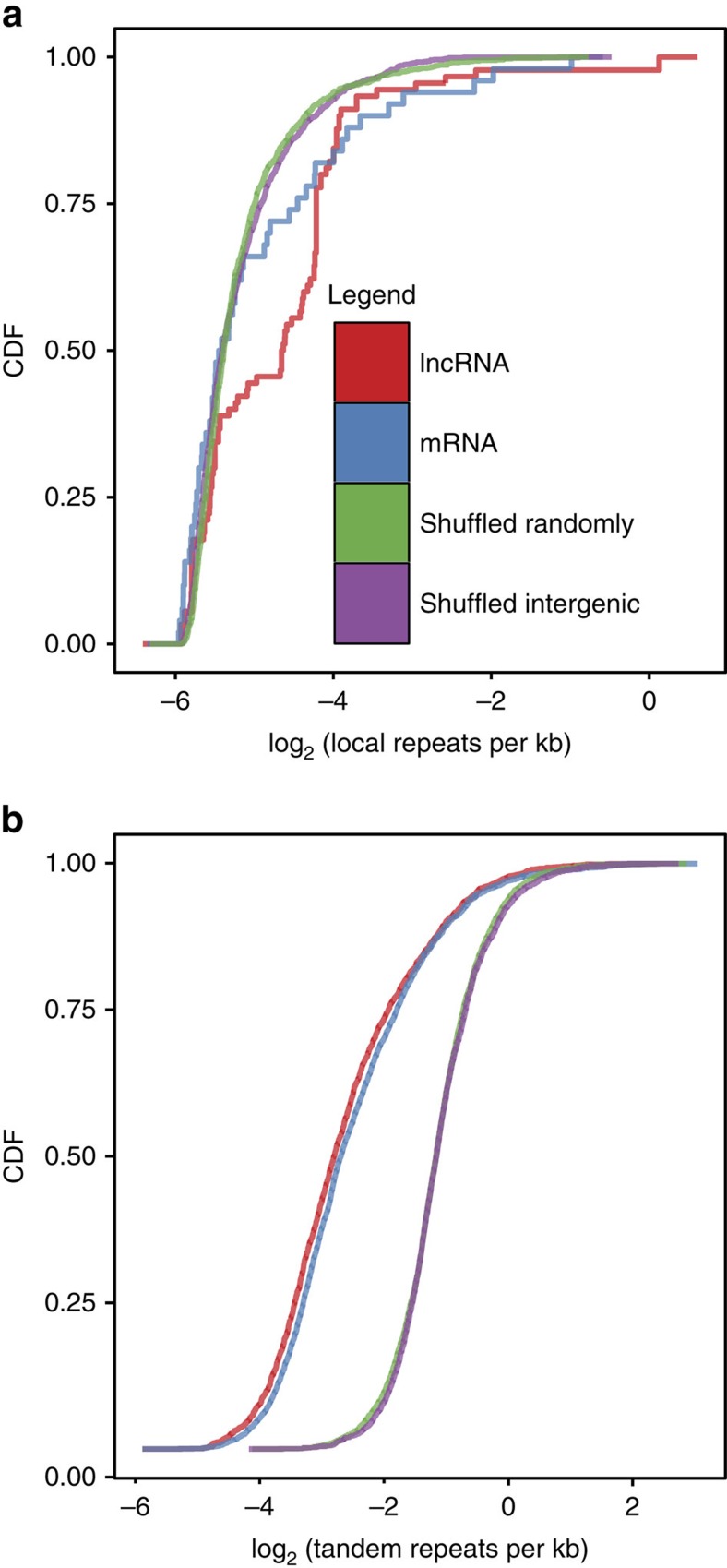
lncRNAs have more local repeats compared with mRNAs. (**a**) Cumulative density plot of local repeats per kb for mRNAs, lncRNAs and the control sets. (**b**) Cumulative density plot of tandem repeats per kb for mRNAs, lncRNAs and the control sets. CDF, cumulative distribution function.

After establishing the locus length-normalized counts (see Methods for details) of LRs and TRs in each gene, we compared the distributions and properties of LRs and TRs between lncRNAs and mRNAs. We found that lncRNA genes have significantly more LRs per kb than protein-coding genes (Mann–Whitney *U*-test, *P*-value<2.2e-16; Fig. 1a, Supplementary Fig. 1B). Although lncRNAs harbouring LRs have on average ∼25 LRs per 100 kb; mRNAs harbouring LRs only have ∼6 LRs per 100 kb of genomic sequence—an enrichment of fourfold. Moreover, LRs are still more enriched in lncRNAs when compared with both the genomic and intergenic null distributions (Mann–Whitney *U*-test, *P*-value<2.2e-16). This is in contrast to TEs, which are enriched in lncRNAs but not above average genomic background^7^. The mean and median lncRNA LR length were 1,813 and 436 bp, respectively. For mRNAs, we observed a mean and median LR length of 471 and 162 bp, respectively. The higher average LR length was explained by the presence of a small number of very long LRs (>5 kb) in both mRNAs and lncRNAs. No difference in the GC content of LRs between mRNAs and lncRNAs was observed: mean and median LR GC content were 48.88 and 50.35% bp for lncRNAs and 48.31 and 49.05% for mRNAs.

In contrast, both lncRNAs and mRNAs have a similar number of TRs per kb (Fig. 1b and Supplementary Fig. 1C; Mann–Whitney *U*-test, *P*-value=0.41), which is significantly less than the genomic background (Mann–Whitney *U*-test, *P*-value<2.2e-16). The mean and median lncRNA TR length were 121 and 48 bp, respectively. For protein-coding genes, we observed similar TR lengths with an average of 107 bp and a median of 46 bp. Similar to LRs, no difference in the TR GC content was observed between lncRNAs and mRNAs: average and median GC content of 31.89% and 31.25% for lncRNAs compared with 32.34% and 31.16% for mRNAs, respectively.

Taken together, our analysis suggests that lncRNAs have a propensity to harbour significantly more LRs but not TRs when compared with mRNAs and intergenic space.

### The *FIRRE* locus harbours several LRs

**Figure 2:**
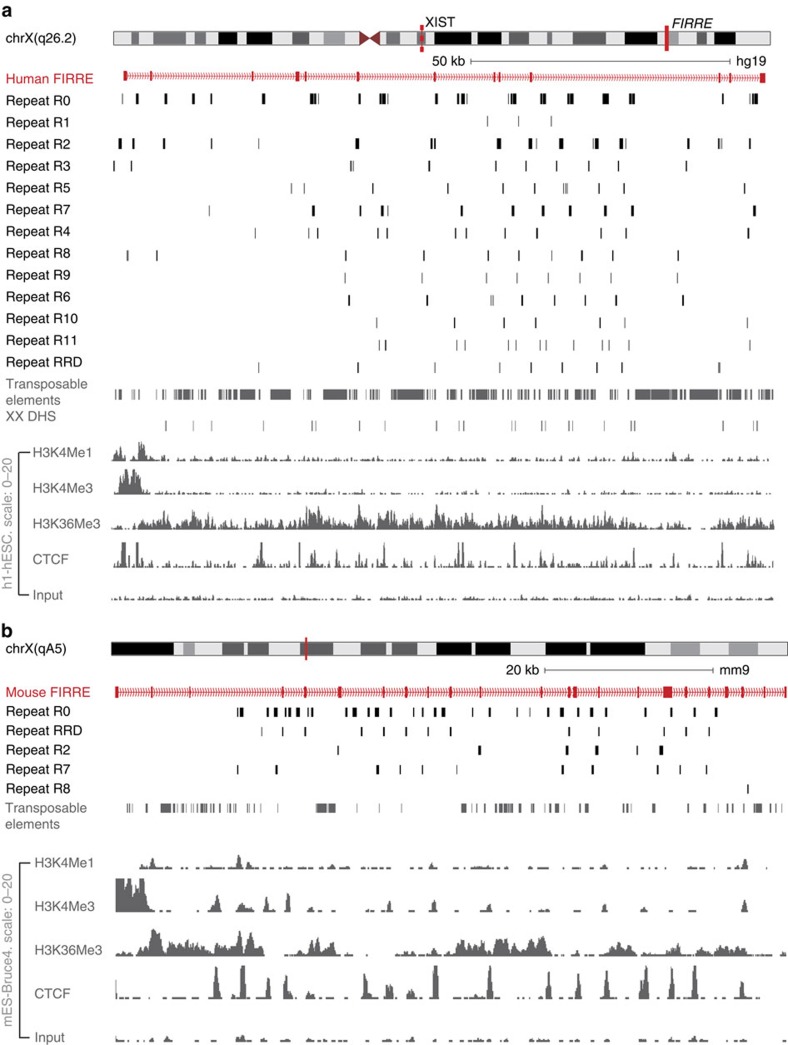
Genomic map of the human and mouse FIRRE locus. (**a**) University of California, Santa Cruz (UCSC) browser screenshot showing the human *FIRRE* locus with all the novel local repeats, transposable elements, histone modifications and transcription factors like H3K4Me1, H3K4Me2, H3K4Me3, H3K36Me3 and CTCF in human embryonic stem cells (hESCs). (**b**) UCSC browser screenshot showing the mouse *FIRRE* locus with the local repeats conserved between human and mouse, transposable elements, histone modifications and transcription factors like H3K4Me1, H3K27Ac, H3K4Me3, H3K36Me3 and CTCF in mouse embryonic stem cells (mES-Bruce4).

We and other groups have also shown that the *FIRRE* locus is highly repetitive with many interesting properties, potentially mediated by repeats^28^,^40^,^42^. Our LR identification pipeline above uncovered 12 new LRs (in addition to RRD) in *FIRRE* (Fig. 2a). Collectively, we named these repeats R0–R11 and RRD. These LRs ranged in size from 67 bp (R1) to 804 bp (R2), with a median of 167 bp.

*FIRRE* LRs occur as few as 3 times (R1) and as many as 40 (R0) times in the human genome with a median of 12 occurrences. Out of all these LRs, eight occurred only in the *FIRRE* locus, whereas R0, R2, R3, R4 and R7 were predominantly found in the *FIRRE* locus with a few instances in other regions in the genome. However, these repeats never occur in the same manner as in the *FIRRE* locus: they are shorter than the instances found within the locus and more than 250 kb apart from each other (Supplementary Table 1). Overall, at least 80% of the 13 LRs occur within *FIRRE* and rarely elsewhere in the genome (Supplementary Fig. 1D). Notably, the RRD repeat is the only LR that consistently overlapped with the FIRRE exons, comprising 7 out of the 13 exons in humans.

We next compared the repeat structure between the syntenically conserved human and mouse *Firre* loci. We used the library of 13 LRs (R0–R11 and RRD) that we identified in the human *FIRRE* locus to map to the syntenic mouse *Firre* locus using RepeatMasker. We were able to detect five human *FIRRE* LRs (R0, R2, R7, R8 and RRD) that mapped to the mouse *Firre* locus (Fig. 2b). The repeat structure in mouse was similar to humans, and most of the LRs overlapped intronic regions in the mouse locus. In addition, similar to its human counterpart, the mouse RRD is the only *Firre* LR that overlaps with the exons (11 out of the 23 exons).

### *FIRRE* LRs diverge between primate and rodent lineages

We next traced the evolutionary properties of the *FIRRE* locus and the LRs throughout the mammalian clade. We performed all pairwise alignments of the human *FIRRE* locus and each LR across chimpanzee, orangutan, rhesus macaque, mouse, rat, cat, dog, rabbit, cow and horse species. To ensure diversity in the mammalian order, we selected four primate species (chimpanzee, orangutan, rhesus macaque and human), two rodent species (mouse and rat) and five out-group species (cat, dog, rabbit, cow and horse).

We performed a multiple sequence alignment (MSA) using MAFFT^48^ in order to compare each *FIRRE* LR across different organisms (Supplementary Fig. 2). Surprisingly, the median sequence identity within a species for a given repeat was highly similar (RRD: 93% human, 91% mouse; R0: 85% human, 87% mouse). In sharp contrast, the median sequence identity was much lower across species of primate and rodent orders (R0: human–mouse 58%; RRD: human–mouse 65%; Supplementary Table 2). Thus, we were able to identify orthologous *FIRRE* loci in several mammalian genomes, all of which contain multiple LRs. Although LR sequence identity is high within the species of the same order, they share little sequence conservation across species of different orders.

**Figure 3:**
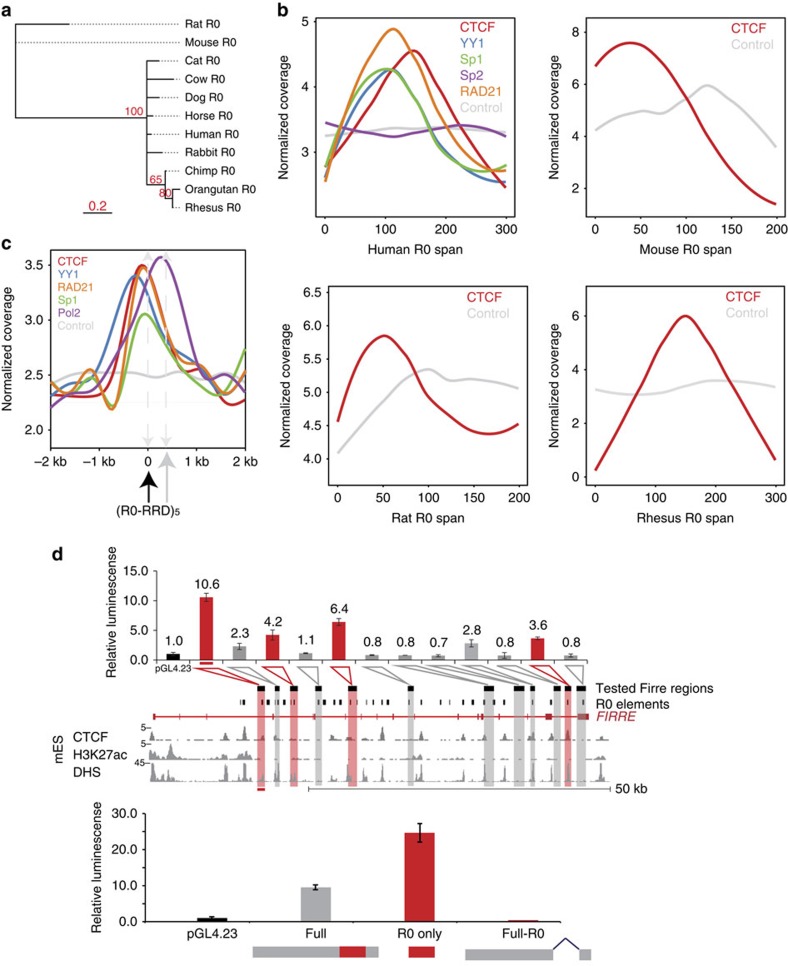
Various transcription factors like CTCF and YY1 specifically bind Repeat R0 in the *FIRRE* locus. (**a**) Phylogenetic tree of local repeat R0 from mammals of different orders. (**b**) ChIP-Seq coverage across repeat R0 gene body for CTCF and input in human, mouse, orangutan, chimpanzee, rhesus macaque and rat. For humans, additional coverage of YY1, RAD21, Sp1 and Sp2 is also shown. (**c**) ChIP-Seq coverage across a 2-kb window centred at the repeat R0 showing RRD a few hundred bps downstream of R0 for CTCF, YY1, Pol2, Sp1, RAD21 and input from the ENCODE consortium data sets. (**d**) Relative luminescence is represented normalized to the pGL4.23 empty vector (black). Each construct was tested in triplicate with standard error of mean (s.e.m.) represented by the error bars. (Top) Several fragments of the *Firre* locus act as enhancers in a luciferase assay. Fragments with greater than threefold increase are highlighted in red. The corresponding *Firre* genomic locus of each fragment is indicated below. In addition, epigenetic marks—H3K27Ac and DNAse Hypersensitivity (DHS) tracks from publically available ENCODE data across the *Firre* locus—are depicted. (Bottom) R0 is required for enhancer activity. The full fragment with the greatest induction (grey), the R0 repeat alone (red) and the fragment with the R0 repeat removed were tested in triplicate (relative luminescence values 9.5, 24.5, 0.4, respectively).

The consensus phylogenetic tree for *FIRRE* (see Methods for details) revealed a distinction between rodents and other mammalian orders, not only for the locus (Supplementary Fig. 2A) but also for the individual repeats: R0–R11 (Fig. 3a and Supplementary Fig. 3A) and RRD (Supplementary Fig. 2B, will be discussed below). These data demonstrate that both the *FIRRE* locus and its LRs have undergone an evolutionary divergence somewhere between rodents and other mammalian orders. It is possible that these LRs have evolved via non-allelic gene conversion, perhaps similar to the mechanism to the repeat ISX that has evolved to have roX-binding sites on the X chromosome in *Drosophila* through non-allelic gene conversion^49^.

### CTCF and RAD21 bind at R0 across multiple species

As all LRs, except for RRD, occur within the intronic sequences of *FIRRE*, they may function as DNA regulatory elements. We hypothesized that some of these repeats may influence the localization of TFs or chromatin regulators. To this end, we investigated a wealth of TF and CTCF chromatin immunoprecipitation sequencing (ChIP-Seq) data from the ENCODE consortium. It has previously been reported that CTCF is enriched in the *FIRRE* locus, thus we wanted to explore CTCF binding in the context of dozens of other DNA-binding events in the locus (Supplementary Fig. 3B)^27^,^28^,^29^,^30^,^40^.

First, we downloaded the raw reads and mapped them to the human genome using the short read aligner Segemehl^50^, taking special consideration of the multi-mapping reads (see Methods for details). Next, we determined the enrichment of the TF/CTCF ChIP-Seq signal over the genomic background (input) across all instances of the repeat sequence and compared this enrichment to the enrichment of randomly shuffled instances (see Methods for details).

We found that CTCF is specifically enriched at R0 in the *FIRRE* locus in human embryonic stem cells (hESCs; Poisson test: *P*-value<0.001; Fig. 3b,c and Supplementary Fig. 3B). Surprisingly, we were unable to identify a canonical CTCF motif at these binding sites (using CTCF motif from JASPAR vertebrate data set^51^ and PoSSuM^52^ motif matching software, cutoff 1e-6). We were able to identify partial binding motifs but none that passed multiple hypothesis correction (false discovery rate (FDR)<0.05). Consistent with the R0 recruitment of CTCF, the known binding partner RAD21 in the cohesion complex^53^,^54^ is also specifically enriched at R0 (Poisson test: *P*-value<0.001; Supplementary Fig. 3B).

We also did a more exhaustive search to find other TFs interacting with *FIRRE* LRs. First, we mapped all TF motifs from the JASPAR database^51^ to these repeat elements using PoSSuM (cutoff of 1e-6)^52^ and found that that R0 has motifs for several TFs: E2F3, ETS1, SP1, SP2, KLF5 and YY1. To determine if the LRs are indeed bound by these TFs, we analysed publicly available ChIP-Seq data for these factors. We were able to obtain ChIP-Seq data sets from the ENCODE consortium for the enriched motif factors YY1, SP1 and SP2 in hESCs. Implementing a similar analysis to CTCF ChIP (above), we observed that YY1 and SP1 binding are both enriched in R0 in hESCs (Poisson test: *P*-value<0.001; Fig. 3b,c), consistent with our motif analysis. SP2 was not enriched in ChIP data sets at R0 despite the presence of the motif (Fig. 3b). To determine if the YY1–RAD21–CTCF–SP1 complex that is bound at R0 in hESCs is also found in other cell types, we investigated ENCODE data for these marks in GM12878 and NHLF cells. We discovered that these TFs were bound specifically at R0 in both cell types (Supplementary Fig. 3B).

As R0 is conserved across mammals, we next compared the binding of YY1, CTCF, RAD21 and SP1 at R0 across multiple mammals, for which ChIP-Seq data were available. We were limited to CTCF binding for the deepest evolutionary analysis with data sets representing mouse, rhesus macaque and rat in addition to humans. We looked for enrichment of CTCF within the *Firre* locus in mouse embryonic stem cells and hepatocytes from rhesus macaque and rat. In all cases, CTCF was highly enriched in the *Firre* locus with multiple peaks, all of which corresponded to the conserved R0 LR (Poisson test: *P*-value<0.001, Fig. 3b).

Thus, despite the large sequence divergence of R0, it has a conserved property of recruiting CTCF. In humans, we also observed that R0 recruits YY1, Sp1 and RAD21 in addition to CTCF; in the case of CTCF, the recruitment appears independent of consensus DNA-binding motifs. Moreover, our analyses show that CTCF is specifically and significantly enriched at R0 in multiple species.

### R0 elements may function as enhancer elements

CTCF is a well-characterized factor involved in chromosome looping, such as promoter/enhancer interactions^55^,^56^,^57^. Based on most LRs residing in the intronic regions, we hypothesized that there could be DNA regulatory elements harboured in R0 or in other intronic repeats. To test this hypothesis, we amplified fragments of the *Firre* locus to screen for DNA enhancer functions.

First, we designed primers to amplify 1–2 kb fragments tiling the entire 72 kb mouse *Firre* locus. However, because of the repetitive nature of this locus, we were able to successfully amplify only 12 fragments (spanning ∼53 kb region in the *Firre* locus). Importantly, these 12 fragments captured every intronic LR at least once (Supplementary Table 3). The fragments housed 19 copies of various intronic LRs: 14 R0 copies, 2 R3 copies, 2 R8 copies and 1 R10 copy. Briefly, we cloned these fragments upstream of a firefly luciferase gene driven by a minimal promoter (Supplementary Fig. 3C) and transfected 3T3 cells in triplicates. We measured the fold increase in luminescence for each plasmid containing a tested fragment relative to the luminescence observed for the empty vector containing only the minimal promoter sequence (see Methods for details). An increase of relative luminescence indicates an inherent ability of the tested DNA fragment to enhance the expression of the luciferase reporter, as compared with baseline expression from the minimal promoter alone.

We observed four fragments with an ability (>3-fold) to enhance expression of the luciferase reporter gene (Fig. 3d). We found that R0 is the only intronic LR present in any of the four positive hits. Conversely, all the fragments without R0 failed to show enhancer activity. However, we also saw that some fragments failed to show enhancer activity despite having the R0 sequence. Overall, our results suggested that the observed DNA regulatory activity could reside adjacent to or within the intronic LR R0. Importantly, we observed a CTCF signal in all R0 instances found in the four positive fragments, highlighting the potential functional relevance of CTCF enrichment at R0 repeats and its DNA regulatory activity.

We next wanted to test whether R0 is required for the observed regulatory activity. To that end, we generated three luciferase reporter gene constructs derived from our DNA fragment with the greatest relative luminescence. The constructs contained either the entire DNA fragment, the R0 sequence alone or the DNA fragment with the R0 sequence excised cloned upstream of the minimal luciferase promoter (Fig. 3d). The R0 repeat alone was able to enhance the expression of the luciferase reporter gene (25-fold), even more than the full-fragment construct (10-fold). Furthermore, the excision of the R0 element from the full-fragment construct ablated any enhancer-like activity (Fig. 3d). These results suggest that R0 and potentially CTCF binding are important enhancer-like regulatory factors.

### RRD functions as a nuclear localization signal

After detecting specific binding of important TFs at the intronic repeats, we were intrigued by whether repeats might also play a role at the RNA level. We first investigated RRD as it is the only repeat that is exclusively in the exons of the Firre transcript. We have previously discovered that overexpression of an isoform of Firre without RRD results in the translocation of Firre transcripts into the cytoplasm^27^. Therefore, we wanted to test whether RRD is sufficient to localize any RNA in the nucleus.

**Figure 4:**
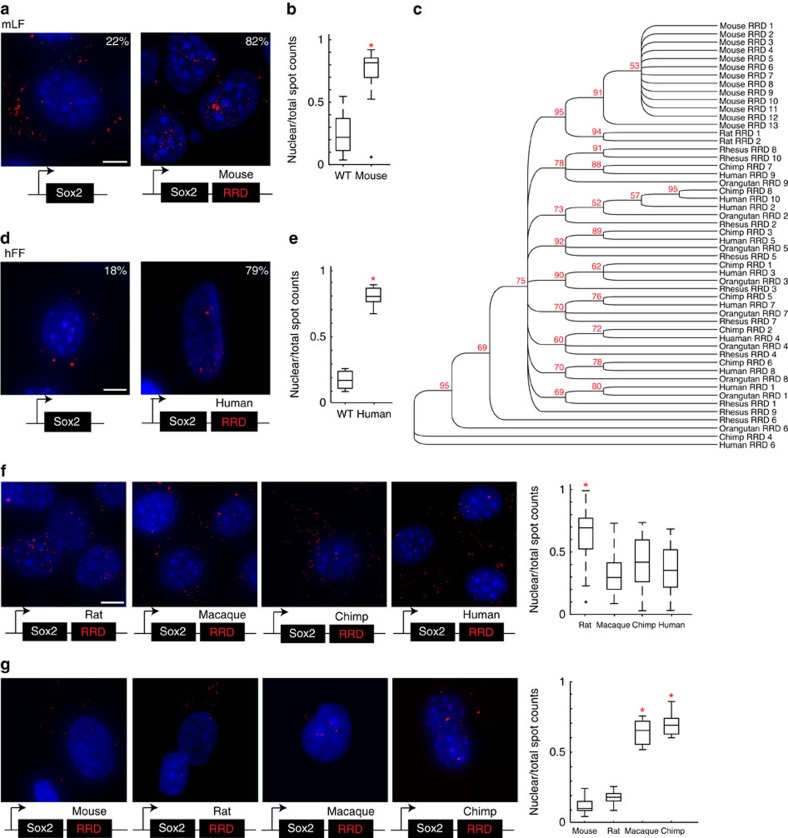
Evolution of the sequence and role of repeat RRD. (**a**) Viral overexpression of Sox2 and Sox2+mouse RRD in mLFs shown using smRNA FISH. Alexa 594 (‘red') targeting the Sox2 exon. (**b**) MATLAB quantification (StarFISH) of the percentage of Sox2 transcripts (Sox2 and Sox2+mouse RRD) localized in the nucleus in mLFs. (**c**) Phylogenetic tree of local repeat RRD from mammals of different orders. (**d**) Overexpression of Sox2 and Sox2+human RRD in hFFs shown using smRNA FISH as described in **a**. (**e**) Quantification of Sox2 transcripts (Sox2 and Sox2+human RRD) as described in **b**. Sox2 nuclear localization percentages are included in **a** and **d**. (**f**,**g**) Overexpression of Sox2 constructs appended with RRDs from different species in mLFs (**f**) and hFFs (**g**). MATLAB quantification performed as in **b**,**e**. Scale bar, 10 μm (**a**,**f**). Scale bar, 15 μm (**d**,**g**). WT, wild type.

To determine if RRD is sufficient to localize transcripts in the nucleus, we have made constructs, in which the consensus RRD sequence is appended to the 3′ of an otherwise cytoplasmic mRNA, Sox2. We chose to perform these overexpression experiments in mouse lung fibroblasts (mLFs) because they do not endogenously express Sox2. Mouse Sox2 was cloned into a lentiviral overexpression vector, which we made and termed ‘lincXpress' (Supplementary Fig. 4A,B). Subcellular localization analysis of Sox2 after overexpression was performed both by fractionation and single-molecule RNA fluorescence *in situ* hybridization (smRNA FISH) with exonic probes conjugated to Alexa 594 targeting Sox2 as described^58^,^59^. We first overexpressed Sox2 alone in mLFs and observed that ∼80% of total number of Sox2 transcripts localize in the cytoplasm (Fig. 4a). For every condition, we counted more than 40 nuclei using StarFISH^50^. We then repeated the same experiment but with mouse RRD added to the 3′ end of Sox2. We observed that upon the addition of the mouse RRD to Sox2, ∼80% the total number of Sox2 transcripts localized in the nucleus (*t*-test, *P*<7.10e-9; Fig. 4a,b). We further verified the distributions of Sox2 transcripts by biochemical fractionation of nuclear and cytoplasmic compartments upon inspecting that the expression levels were comparable by quantitative reverse transcription–PCR (qRT–PCR; Supplementary Fig. 4C,D). These results suggest that mouse RRD serves as a sufficient signal to localize an RNA in the nucleus in the same species.

Similar to the *FIRRE* locus, the R0 repeat and RRD also shows a distinct evolutionary split between rodents and primates (Fig. 4c). The mouse and rat RRDs cluster separately from the primate RRDs, and bootstrapping shows this is highly significant (95/100 permutations). Moreover, within the primate RRDs, no obvious sub-clusters are readily visible (Fig. 4c and Supplementary Fig. 2B), as all the primate sequences seem to have converged and cannot be distinguished easily in the phylogenetic tree (Fig. 4c); whereas, the rodent and other mammalian lineages are highly divergent in sequence identity.

Based on this evolutionary divergence, we next wanted to test whether the nuclear localization property was a unique feature of the mouse RRD or an evolutionarily conserved phenomenon across species. To that end, we made another Sox2 construct by appending the consensus sequence of human RRD to the 3′ end of Sox2 (Supplementary Fig. 4A,B). We then overexpressed Sox2 alone or Sox2 with human RRD at similar levels (Supplementary Fig. 4E) in human foreskin fibroblasts (hFFs) and investigated their subcellular localization as described above. Similar to mLFs, hFFs also do not endogenously express Sox2. Our analyses showed that Sox2 alone resulted in a mostly cytoplasmic localization; whereas, human RRD significantly altered the distribution of Sox2 RNAs to be more nuclear (*P*<3.94e-009; Fig. 4d,e). Together, these results showed that RRD acts a nuclear localization signal in both mouse and human.

### RRD is a species-specific nuclear localization signal

Although the LRs in the *FIRRE* locus are syntenically conserved in human and mouse, they share only ∼68% sequence identity (Supplementary Table 1). Yet, they seem to share the same function of binding to multiple protein partners. Based on the ability of orthologous RRDs to sufficiently retain an otherwise cytoplasmic transcript in the nucleus, we wanted to further investigate if this function is conserved across other species (as was CTCF binding at R0).

To this end, we overexpressed Sox2 appended at its 3′ end with the consensus RRD sequences from four other species in addition to mouse: rat, macaque, chimp and human as described above (Supplementary Fig. 4B) in mLFs. We performed a similar smRNA FISH analysis and checked expression levels by qRT–PCR (Supplementary Fig. 4C) as described above.

Similar to the case with the mouse RRD, we observed an almost exclusively nuclear localization of Sox2 appended with rat RRD but not with macaque, chimp or human RRDs. Upon counting ∼300 nuclei (together with Sox2 alone and Sox2+mouse RRD), our analyses revealed that the rat RRD also skews the distribution of Sox2 transcripts to be more nuclear: 65% (*P*<4.06e-14; Fig. 4f). In contrast, there is a significant reduction in the number of Sox2 transcripts that localize in the nucleus when macaque, chimp or human RRD is added to the 3' end of Sox2 (28% (*P*<0.0286), 40% (*P*<3.08e-4) and 31% (*P*<0.0068), respectively; Fig. 4f). We have further verified the distributions of transcripts by biochemical fractionation of nuclear and cytoplasmic compartments (Supplementary Fig. 4D). Collectively, these results suggest that in mLFs, the rodent RRD sequences are sufficient to localize Sox2 in the nucleus; whereas, the primate lineage RRD sequences do not have an effect on the distribution of Sox2 subcellular localization.

Upon observing that similar to the mouse RRD in mouse cells, human RRD is able to skew the distribution of Sox2 transcripts to be more nuclear in hFFs, we performed the overexpression experiments with mouse, rat, macaque and chimp RRDs in hFFs (Supplementary Fig. 4B). We counted ∼300 nuclei and observed a reciprocal effect: the mouse and rat RRDs do not result in nuclear localization of Sox2; whereas macaque, chimp and human RRDs significantly alter the distribution of Sox2 RNAs to be more nuclear (*P*<1.49e-006, *P*<3.37e-007, *P*<3.94e-009) by smRNA FISH (Fig. 4g). Furthermore, we have confirmed that the difference in the distribution of transcripts is not due to a difference in the expression levels of the respective RNA species (Supplementary Fig. 4E). Overall, our results show a divergence in sequence evolution between the rodent and primate lineages while maintaining a shared ability to ectopically serve as a nuclear localization signal.

### hnRNPU might influence RRD-based nuclear localization of RNAs

The shared role of RRD to be sufficient as a nuclear localization signal raised the question of which protein factors may be responsible. We have previously found that heterogeneous nuclear ribonucleoprotein U (hnRNPU) binds Firre via RRD, and depletion of hnRNPU results in mislocalization of Firre transcripts into the cytoplasm in HEK293s and HeLa cells^27^. Furthermore, the loss of hnRNPU also affected the co-localization of Firre with its trans-chromosomal targets in the nucleus^27^. This lead us to further investigate the binding properties of hnRNPU and RRD with respect to nuclear localization.

**Figure 5:**
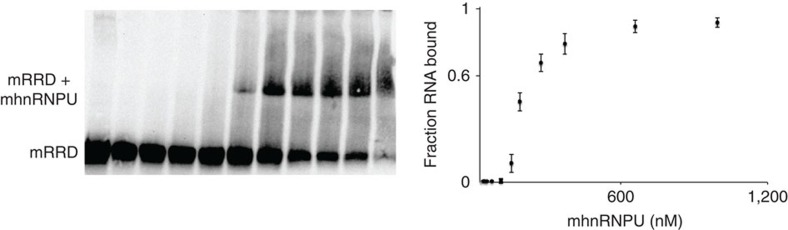
hnRNPU binds RRD with high affinity. Electrophoretic mobility shift assay showing mouse RRD and mouse hnRNPU (on the left), quantified by the curve on the right. Kd: 200±50 nM, with hnRNPU 5 nM–1.5 μM.

First, we determined the binding affinities of mouse hnRNPU:mouse RRD and human hnRNPU:human RRD interactions. Briefly, we purified human and mouse hnRNPU proteins using a BioEase tag affinity purification followed by AcTEV protease elution (see Methods for details). We tested the binding affinities via electrophoretic mobility shift assay using *in vitro* transcribed human and mouse RRD RNA sequences. Keeping the RNA concentration constant at 25 nM (∼28 ng), we titrated in purified hnRNPU starting from 5 nM to 1.5 μM. We found that the dissociation constant (Kd) of the mouse RRD and mouse hnRNPU interaction is 200±50 nM (Fig. 5). Similarly, the affinity of the interaction between human RRD and human hnRNPU is 180±25 nM (Supplementary Fig. 5A). In sharp contrast, the cross-species interaction of mouse RRD and human hnRNPU had a significantly lower affinity (Supplementary Fig. 5B), indicating that there is a species-specific interaction between hnRNPU and RRD.

**Figure 6:**
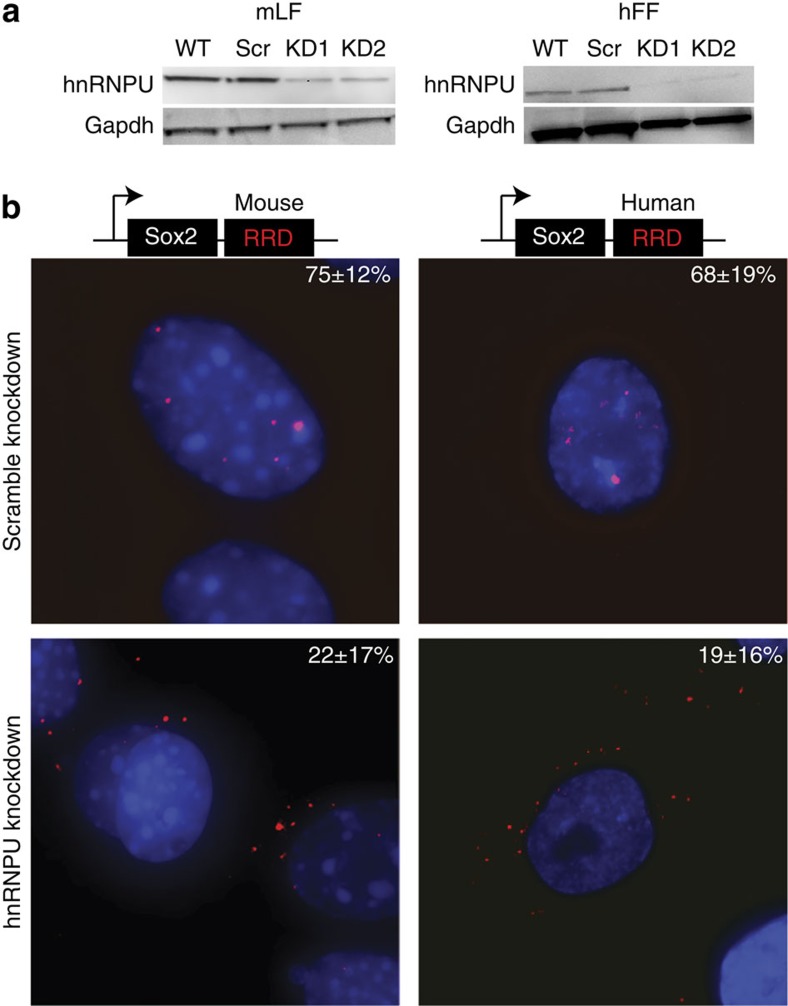
hnRNPU affects RRD-dependent nuclear localization. (**a**) hnRNPU was knocked down (KD) in mLFs and hFFs using siRNAs. Scr refers to the scramble siRNA and KD1 and 2 refer to different siRNAs used. Gapdh used as loading control. (**b**) Sox2+mouseRRD and Sox2+human RRD were overexpressed in hnRNPU KD conditions in mLFs and hFFs, respectively. smRNA FISH using Alexa 594 (‘red') probes targeting the Sox2 exon. Sox2 nuclear localization percentages are included. WT, wild type.

Based on the hnRNPU and RRD interaction, we hypothesized that hnRNPU might play a critical role for how RRD affects Sox2 distribution. To test this hypothesis, we performed RNA interference (RNAi)-mediated knockdown of hnRNPU in mLFs and hFFs, using targeting and non-targeting (as negative controls) siRNAs (Fig. 6a,b). After 48 h, we repeated the Sox2+mouse RRD and Sox2+human RRD transductions, respectively, along with Sox2 alone transduction in both cell lines.

We found that the knockdown of hnRNPU had a dramatic effect on the nuclear localization of Sox2+mouse RRD in mLFs and Sox2+human RRD in hFFs but not on the cytoplasmic distribution of Sox2 alone (Fig. 6b and Supplementary Fig. 6C), suggesting that hnRNPU could play a role in keeping transcripts with RRD in the nucleus. We are aware of the role of hnRNPU in nuclear organization; therefore, the alteration in the nuclear to cytoplasmic distribution of these transcripts can be an indirect effect, caused by change of the overall organization in the sub-nuclear territories bound by hnRNPU. Together, our data suggest that the LRs and the *Firre* locus have evolved, both as DNA and RNA, concordantly and show conserved functions despite large evolutionary drift.

## Discussion

It is becoming increasingly clear that the repetitive elements are essential to genome function. For example, there are now well-documented examples that show how TEs influence the expression and network dynamics of lncRNAs, as exemplified by the HERVH elements enriched in lncRNAs in stem cells^7^,^12^,^49^,^60^. Moreover, these TE-regulated lncRNAs—such as linc-ROR—play important functional roles in establishing and maintaining pluripotency^61^,^62^,^63^. Here, we describe that LRs can also represent functional domains, specifically within the *Firre* locus. More globally, LRs are highly enriched in lncRNAs compared with the rest of the genome, raising the possibility that they might constitute important lncRNA domains.

The only LR in the *Firre* locus that only overlaps with exons is RRD. This could perhaps suggest that RRD represents a functional domain within the mature Firre transcript. Consistent with this notion, we identified two inter-related RNA-based functions of RRD. First, RRD serves as a nuclear localization signal that is necessary and sufficient to localize an otherwise cytoplasmic mRNA in the nucleus. Moreover, RRD serves as a conserved nuclear localization signal in both primate and non-primate lineages. Similarly, RRD has a conserved property to bind to hnRNPU, which is required for proper nuclear localization of Firre in both human and mouse cells. Our results are reminiscent of the Xist lncRNA, which loses its proper localization on the inactive X upon hnRNPU knockdown^64^. Collectively, our findings demonstrate that RRD comprises a novel RNA nuclear localization signal. This raises the question of whether LRs in other lncRNAs might also function as localization sequences or aid the formation of distinct subcellular domains.

In addition to the exonic LR, we also investigated the repeats in the intronic regions of *FIRRE*. One of these LRs, R0, shows an evolutionarily conserved CTCF binding in primates and rodents, in addition to critical chromatin factors, such as YY1 and RAD21. Interestingly, although previous studies have identified CTCF-binding motifs^65^,^66^, repeat R0 does not seem to contain the canonical CTCF-binding motif. This suggests a couple of possible alternatives: (i) the weaker and non-significant (after multiple hypothesis correction) CTCF motifs could still possibly recruit CTCF; (ii) CTCF could interact with other sequence-specific factors at these sites, but are not common for CTCF localization genome wide; (iii) CTCF recognizes the structure of the R0 repeat in RNA form; (iv) CTCF-binding sites at R0 could reflect a higher order chromosomal interaction, in which these sites are in proximity to other loci canonically bound by CTCF. The latter possibility is intriguing because we have previously found Firre to form three-dimensional interactions with multiple loci^27^.

Interestingly, several epigenetic features within the *FIRRE* locus have been reported to be regulated in a sex-specific manner^28^,^40^,^41^,^67^,^68^. For example, the epigenetic status of the *FIRRE* locus can predict the correct sex in nonmalignant cell types^41^. In fact, it is the only other region on the X chromosome besides *XIST* that shows sex-specific epigenetic regulation^68^, suggesting that *FIRRE* might have a sex-specific role. Beyond sex-specific differences, we also note significant evolutionary differences in the *FIRRE* locus yet with conserved properties (CTCF binding to R0 and RRD nuclear retention).

Taken together, our data demonstrate that LRs can serve as functional RNA and DNA domains. This raises the question of how many other exonic LRs represent functional domains, such as localization signals. Although many nuclear lncRNAs have been studied in-depth, the specific domains that determine the localization properties of these RNAs remain unresolved. Thus, LRs may represent molecular RNA modules for specific functionalities, ranging from protein interactions to sub-cellular localization. Consistent with this notion LRs are enriched in lncRNAs. Examination of these repetitive sequences will require additional computational and experimental analyses but will provide much needed first steps towards understanding how RNA-based domains function and may reveal potential common families similar to those of protein-coding genes.

## Methods

### Pipeline for surveying novel LRs and TRs

The coding genes are, on average, longer than lncRNAs (Supplementary Fig. 1A), which could potentially lead to artificial differences in the distributions of repeats. To control for this difference, we sampled equal numbers of mRNAs and lncRNAs with similar length distributions (Supplementary Fig. 1A). We further estimated background rates of LRs and TRs by taking similar-sized windows of each annotation followed by 100 permutations of randomly selected windows located in genomic and intergenic regions. From these permutations, we separately sampled sequences equal to the number of lncRNAs in our catalogue for both controls (genomic and intergenic), making sure that the length distributions of the sampled windows and lncRNAs are similar.

For each gene, we masked out the TEs annotated in the RepeatMasker file from the University of California, Santa Cruz (UCSC) genome browser. Next, we used RepeatScout to *de novo* find repeats in this repeat-masked gene sequence. To get only the LRs, TRF was used to remove any TRs from the set discovered by RepeatScout. Finally, to get all instances of a given LR, we mapped our LR catalogue to the human genome using RepeatMasker. As RepeatMasker uses a BLAST programme as the basis of comparing sequences sometimes there are short overlaps (5–10 bp) between regions annotated as LRs and TEs. Separately, we used TRF to find all TRs in the masked gene sequence and compile a catalogue of TRs.

### Statistical tests

The lncRNA annotation file was shuffled in two ways to get separate control sets. In the first case, the annotation file was shuffled to allow the new regions to be anywhere in the genome (shuffled). In the second case, the annotation file to only fall in unannotated intergenic regions of the genome in order to compare the repeat distribution of lncRNAs with other random intergenic regions (shuffled intergenic). LRs and TRs were found as described above in both these sets and the numbers in each set were compared separately with lncRNAs and mRNAs. Mann–Whitney test was used to compare the number of repeats in any two sets.

### Multi-mapping reads

Although analysing interactions at repetitive regions, it is very important to carefully interpret multi-mapping reads. We required Segemehl to allow a large number (100,000) of seed alignments but only output 20 best alignments for each read. Next, in order to count the number of reads mapping to a particular region, we normalized the reads by the number of locations, to which they align. For example, a read mapping to 20 positions in the genome will be counted as 1/20th at each position. Such an approach has been used in several papers previously to analyse reads at repetitive sequences.

### ChIP-Seq analysis

First, we downloaded fastq files of ChIP-Seq reads generated by the ENCODE consortium from UCSC for CTCF, YY1, Sp1, Sp2 and Pol2. Next, we used the short-read mapper Segemehl to map the reads to the genome paying special attention to the multi-mapping reads. The alignments generated by Segemehl were used to plot coverage of the reads over a repeat region as well as compute enrichment over it.

We calculated coverage for the repeat in TF ChIP-Seq and divided it by the coverage of the repeat in the input to obtain the enrichment of a given TF across all instances of the repeat. Next, we calculated a similar enrichment of the TF across random but equal-sized regions of genomic space (100 permutations). Finally, to calculate if the enrichment of the repeat was significantly higher than randomly shuffled sequences, we used a Poisson distribution as the background null model. The average of the enrichment scores for the 100 permutations of the shuffled sequences was used to determine the Poisson model parameter and compared directly to the enrichment computed for the repeat element to obtain a *P*-value.

### Phylogenetic trees

We built a MSA of the input sequences using MAFFT^48^ run with default parameters. Using this MSA, we constructed a phylogenetic tree using a neighbor-joining method^69^. To calculate the confidence for each branch, we used a bootstrapping approach and reported the branches with >50% confidence in the bootstraps.

### Luciferase assay

3T3 cells were plated in a 96-well plate (Corning 3904) at a density of 2.0 × 10^4^ per 100 μl one day before transfection. The minimal promoter construct pGL4.23 (Promega) and the *mFirre* fragment constructs, pKW01-pKW09, pKW12-pKW14 (Supplementary Table 3), were co-transfected along with the Rennila vector pGL4.72 (Promega) in triplicate by Lipofectamine 3000 (Life Technologies) according to the manufacturer's instructions. Media were changed every 24 h following the transfection. Luciferase expression was assayed 72 h post transfection on a BioTek Cytation with the Dual-Glo Luciferase Assay System (Life Technologies, E2920) according to the manufacturer's protocol.

### Cloning

For the luciferase assay, the vector pGL4.23 [luc2/minP] was digested with *Eco*RV for 1 h followed by calf intestinal alkaline phosphatase (NEB M0290) treatment. Fragments spanning the *mFirre* locus were generated by PCR (Phusion-HF; NEB, #M0531) and isolated by size on a 1% agarose gel. Purified products were then treated with T4 Polynucleotide Kinase (NEB, #M0201) followed by PCR Purification (Qiagen) clean-up. Phosphorylated *mFirre* fragments were cloned into the *Eco*RV multiple cloning site of pGL4.23 [luc2/minP] (Promega) in a Quick Ligation reaction (NEB, #2200). Immediately following the ligation, constructs were transformed into chemically competent *E. coli* DH5alpha cells (Life Technologies, #12297016) under ampicillin selection. Successful *mFirre* fragment constructs (pKW01-pKW09, pKW12-pKW14) were verified by sequencing.

lincXpress vector (Supplementary Figure 4A) was made by modifying the pLenti6.3/TO/V5-DEST (Snap Gene) destination vector as described previously^27^. Sox2 was PCR amplified using the Gateway tails as described in the Life Technologies manual. The PCR conditions were: (i) 98 °C for 30 s, (ii) 98 °C for 10 s, (iii) 61.7 °C for 30 s, (iv) 72 °C for 30 s, (v) 72 °C for 5 min and (vi) 4 °C final, with 24 cycles repeating steps 2–4. The purified PCR product was then cloned into the pDONR vector (Life Technologies, #12535-035) in a BP reaction (Life Technologies, #11789020), which was followed by an LR reaction to move Sox2 into the lincXpress backbone (Life Technologies, #11791-043). All these cloning steps were performed according to the instructions in the Gateway cloning manual. The RRDs from each species were cloned at the 3′ end of Sox2 in the lincXpress vector using Gibson Assembly according to the instructions in the manual (NEB, # E2611). The RRDs were PCR amplified using primers with Gibson arms and assembled with the *Kpn*1-linearized and purified Sox2 backbone. For transformations, 20 μl of XL10-Gold cells (Agilent, # 200314) were used. BP reactions were grown on Kanamycin plates and the rest were on Ampicillin plates. LR reactions were grown at 30 °C overnight to prevent recombination. All of the vectors were sequenced through Genewiz, and the verified plasmids were prepared using the maxi-prep kit (Qiagen, #12163). All the primers used for cloning are shown in Supplementary Table 4.

### Cell lines

mLF (American Type Culture Collection (ATCC): CCL-206), hFF and HEK293 (ATCC: CRL-1573) cells were grown in DMEM (Life Technologies), 10% FBS (Life Technologies), 1% Penicillin–Streptomycin (Life Technologies) and 1% L-Glutamine (Life Technologies) at 37 °C at 5% CO_2_. 3T3 cells were cultured according to the ATCC guidelines.

### Viral overexpression

To generate virus from the lincXpress constructs, 95% confluent HEK293 cells were transfected with 7.5 μg of the vector, 22.5 μl of the viral packaging mix (pLP1, pLP2 and pLP/VSVG, 1 μg μl^−1^) and 90 μl of Lipofectamine 2000 (Life Technologies, #11668-027). The media collected from HEK293s after 72 h were prepared according to the ViraPower Lentiviral Expression Systems manual (Invitrogen/Life Technologies) to prepare the final viral particles.

All the transductions were done as described previously in Hacisuleyman *et al*^27^. The media were renewed 24 h after the transduction and kept in the same media for another 24 h. Then, the cells (100,000 cells per well) were split on to the two-well dishes for overnight growth (Nunc Lab-Tek Chambered Coverglass, ThermoScientific/VWR, # 155380) for smRNA FISH. The untransduced controls were used to measure the overexpression levels by qRT–PCR and to confirm that there is no signal for Sox2 in mLFs and hFFs by smRNA FISH.

### Single-molecule RNA fluorescence *in situ* hybridization

The FISH protocol was followed as described previously^27^,^59^. Briefly, mLFs and hFFs were grown overnight in two-chamber slides and fixed with 10% formaldehyde for 10 min at room temperature. The probes targeting and tiling the Sox2 exon were conjugated to Alexa 594 (‘red'). The nuclei were stained with 4,6-diamidino-2-phenylindole (‘blue'). For the overexpression experiments across species, ∼150 cells were counted for each cell line. For each image, 30–35 *z*-stacks were taken with each slice 0.27–0.33 μm. The images were obtained using the Cell Observer Live Cell microscope at the Harvard Center for Biological Imaging.

### RNAi-mediated knockdown of hnRNPU

mLFs and hFFs were reverse-transfected in 12-well plates. Per well, 85,000 cells were plated at the time of transfection. In the knockdown data, KD1 and KD2 refer to different siRNAs. For the mouse hnRNPU: knockdown 1 was performed with Set of 4 Upgrade: ON-TARGETplus mouse Hnrnpu siRNA, Dharmacon/ThermoScientific, LU-051574-01-0002, and knockdown 2 with mouse hnRNPU siRNA Silencer Select, Life Technologies, 4390771. For the human hnRNPU: knockdown 1 was performed with SMARTpool: ON-TARGETplus HNRNPU siRNA, Dharmacon/ThermoScientific, L-013501-00-0005 and knockdown 2 with human hnRNPU siRNA Silencer Select, Life Technologies, 4392420. Lipofectamine RNAiMAX (Life Technologies, #13778030) and siRNA (75 nM final) complexes were prepared in Opti-MEM according to the instructions in the RNAiMAX manual. The complexes were incubated at room temperature for 20–30 min then added on to the cells. The media were changed after 24 h and the cells were transduced with Sox2 or Sox2+mouse RRD or Sox2+humanRRD constructs after 48 h. The amount of knockdown was determined by western blot analysis and qRT–PCR as described previously^27^. The antibodies used for hnRNPU were: human hnRNPU (3G6): SantaCruz sc-32315 (validated and shown on the Santa Cruz website), mouse hnRNPU: Abcam ab20666 (validated by SAF-A Has a Role in Transcriptional Regulation of Oct4 in ES Cells Through Promoter Binding). The primers for hnRNPU are outlined in Supplementary Table 4.

### hnRNPU purification and electrophoretic mobility shift assay

Human and mouse hnRNPU cDNAs were obtained from ThermoScientific (MHS1011-202832408 and MMM4769-202762349, respectively) and cloned into pDONR by BP, then into pcDNA3.2/capTEV-NT/V5-DEST backbone by LR reactions as described above. The capTEV-NT vector has the TEV-Bioease Tag along with V5 and 6XHis tags at the N terminal of the cDNA that is cloned. The primers used to PCR amplify the cDNAs are listed in Supplementary Table 4.

Protein was purified from HEK293 cells, which were grown in 15 cm dishes. The cells were plated 24 h before transfection, and 2 h before transfection growth media were replaced with 12.5 ml antibiotic-free growth media. The cells were then transfected at ∼85% confluence with 90 μl of Lipofectamine 2000 and 40 μg of the hnRNPU construct. Briefly, Lipofectamine and plasmid were diluted in 3.75 ml Opti-MEM separately and incubated at room temperature for 5 min. Then, the two were mixed and incubated for another 20 min at room temperature. The complexes were then added to the cell drop-wise. The media were changed the next day, and the cells were harvested after 48 h.

For the purification, the NativePure Affinity manual was followed. Certain steps were modified to decrease the background protein carry-over. First, instead of Streptavidin Agarose beads, Magnetic MyOne Streptavidin T1 Beads (Life Technologies, #65601) were used. Second, instead of the lysis buffer suggested by the protocol, the cells were lysed by using another lysis buffer: 150 mM KCl, 25 mM Tris-HCl, pH 7.4, 5 mM EDTA, 5 mM MgCl_2_, 1% NP-40, 1 × protease inhibitor, 0.5 mM dithiothreitol, 100 U ml^−1^ RNAseOut. In order to lyse the cells, 1 ml of the outlined lysis buffer was added on the cells and the rotated at 4 °C for 30 min. The cells were then scraped, pipetted up and down ten times, and centrifuged at 12,000*g* for 30 min at 4 °C. Finally, instead of the advised 400 U of AcTEV Protease, 250 U were used at the final protein elution step. The fractions from each step were collected; upon measuring the protein concentrations in each, they were run on an SDS–polyacrylamide gel electrophoresis gel.

Human and mouse RRDs were *in vitro* transcribed as described previously^27^. The purified RNA and proteins were then used in electrophoretic mobility shift assays, which were performed using the LightShift Chemiluminescent RNA electrophoretic mobility shift assay kit (ThermoScientific, #20158). For each reaction, 30–50 nM RNA was used, and the protein was titrated starting at 5 nM up to 1.5 μM. The conditions suggested in the manual were modified: the reactions were performed in 10 mM MgCl_2_, 1 mM dithiothreitol and 0.1 μl RNAseOut, and the transfer was performed using the semi-dry method.

## Additional information

**How to cite this article:** Hacisuleyman, E. *et al*. Function and evolution of local repeats in the *Firre* locus. *Nat. Commun.* 7:11021 doi: 10.1038/ncomms11021 (2016).

## Supplementary Material

Supplementary Information Supplementary Figures 1-5 and Supplementary Tables 1-4

## References

[b1] de Koning A. P., Gu W., Castoe T. A., Batzer M. A. & Pollock D. D. Repetitive elements may comprise over two-thirds of the human genome. PLoS Genet. 7, e1002384 (2011). 22144907 10.1371/journal.pgen.1002384 PMC3228813

[b2] Wicker T. . A unified classification system for eukaryotic transposable elements. Nat. Rev. Genet. 8, 973–982 (2007). 17984973 10.1038/nrg2165

[b3] Doolittle W. F. & Sapienza C. Selfish genes, the phenotype paradigm and genome evolution. Nature 284, 601–603 (1980). 6245369 10.1038/284601a0

[b4] Athanasiadis A., Rich A. & Maas S. Widespread A-to-I RNA editing of Alu-containing mRNAs in the human transcriptome. PLoS Biol. 2, e391 (2004). 15534692 10.1371/journal.pbio.0020391 PMC526178

[b5] Kelley D. R., Hendrickson D. G., Tenen D. & Rinn J. L. Transposable elements modulate human RNA abundance and splicing via specific RNA-protein interactions. Genome Biol. 15, 537 (2014). 25572935 10.1186/s13059-014-0537-5 PMC4272801

[b6] Kapusta A. . Transposable elements are major contributors to the origin, diversification, and regulation of vertebrate long noncoding RNAs. PLoS Genet. 9, e1003470 (2013). 23637635 10.1371/journal.pgen.1003470 PMC3636048

[b7] Kelley D. & Rinn J. Transposable elements reveal a stem cell-specific class of long noncoding RNAs. Genome Biol. 13, R107 (2012). 23181609 10.1186/gb-2012-13-11-r107 PMC3580499

[b8] Arkhipova I. R. . Genomic impact of eukaryotic transposable elements. Mob DNA 3, 19 (2012). 23171443 10.1186/1759-8753-3-19 PMC3520738

[b9] Piriyapongsa J. & Jordan I. K. A family of human microRNA genes from miniature inverted-repeat transposable elements. PLoS ONE 2, e203 (2007). 17301878 10.1371/journal.pone.0000203 PMC1784062

[b10] Devor E. J., Peek A. S., Lanier W. & Samollow P. B. Marsupial-specific microRNAs evolved from marsupial-specific transposable elements. Gene 448, 187–191 (2009). 19577616 10.1016/j.gene.2009.06.019 PMC2788610

[b11] Feschotte C. Transposable elements and the evolution of regulatory networks. Nat. Rev. Genet. 9, 397–405 (2008). 18368054 10.1038/nrg2337 PMC2596197

[b12] Schmidt D. . Waves of retrotransposon expansion remodel genome organization and CTCF binding in multiple mammalian lineages. Cell 148, 335–348 (2012). 22244452 10.1016/j.cell.2011.11.058 PMC3368268

[b13] Wang T. . Species-specific endogenous retroviruses shape the transcriptional network of the human tumor suppressor protein p53. Proc. Natl Acad. Sci. USA 104, 18613–18618 (2007). 18003932 10.1073/pnas.0703637104 PMC2141825

[b14] Kunarso G. . Transposable elements have rewired the core regulatory network of human embryonic stem cells. Nature Genet. 42, 631–634 (2010). 20526341 10.1038/ng.600

[b15] Warburton P. E. . Analysis of the largest tandemly repeated DNA families in the human genome. BMC Genomics 9, 533 (2008). 18992157 10.1186/1471-2164-9-533 PMC2588610

[b16] Pellegrini M., Marcotte E. M. & Yeates T. O. A fast algorithm for genome-wide analysis of proteins with repeated sequences. Proteins 35, 440–446 (1999). 10382671

[b17] Snider L. . RNA transcripts, miRNA-sized fragments and proteins produced from D4Z4 units: new candidates for the pathophysiology of facioscapulohumeral dystrophy. Hum. Mol. Genet. 18, 2414–2430 (2009). 19359275 10.1093/hmg/ddp180 PMC2694690

[b18] Winter E. E. & Ponting C. P. Mammalian BEX, WEX and GASP genes: coding and non-coding chimaerism sustained by gene conversion events. BMC Evol. Biol. 5, 54 (2005). 16221301 10.1186/1471-2148-5-54 PMC1274310

[b19] Chadwick B. P. DXZ4 chromatin adopts an opposing conformation to that of the surrounding chromosome and acquires a novel inactive X-specific role involving CTCF and antisense transcripts. Genome Res. 18, 1259–1269 (2008). 18456864 10.1101/gr.075713.107 PMC2493436

[b20] Hall L. L. & Lawrence J. B. XIST RNA and architecture of the inactive X chromosome: implications for the repeat genome. Cold Spring Harb. Symp. Quant. Biol. 75, 345–356 (2010). 21447818 10.1101/sqb.2010.75.030 PMC3143471

[b21] Duszczyk M. M., Wutz A., Rybin V. & Sattler M. The Xist RNA A-repeat comprises a novel AUCG tetraloop fold and a platform for multimerization. RNA 17, 1973–1982 (2011). 21947263 10.1261/rna.2747411 PMC3198591

[b22] Jeon Y. & Lee J. T. YY1 tethers Xist RNA to the inactive X nucleation center. Cell 146, 119–133 (2011). 21729784 10.1016/j.cell.2011.06.026 PMC3150513

[b23] Sarma K., Levasseur P., Aristarkhov A. & Lee J. T. Locked nucleic acids (LNAs) reveal sequence requirements and kinetics of Xist RNA localization to the X chromosome. Proc. Natl Acad. Sci. USA 107, 22196–22201 (2010). 21135235 10.1073/pnas.1009785107 PMC3009817

[b24] Wutz A., Rasmussen T. P. & Jaenisch R. Chromosomal silencing and localization are mediated by different domains of Xist RNA. Nature Genet. 30, 167–174 (2002). 11780141 10.1038/ng820

[b25] Zhao J., Sun B. K., Erwin J. A., Song J. J. & Lee J. T. Polycomb proteins targeted by a short repeat RNA to the mouse X chromosome. Science 322, 750–756 (2008). 18974356 10.1126/science.1163045 PMC2748911

[b26] Costas J., Vieira C. P., Casares F. & Vieira J. Genomic characterization of a repetitive motif strongly associated with developmental genes in Drosophila. BMC Genomics 4, 52 (2003). 14675495 10.1186/1471-2164-4-52 PMC327093

[b27] Hacisuleyman E. . Topological organization of multichromosomal regions by the long intergenic noncoding RNA Firre. Nat. Struct. Mol. Biol. 21, 198–206 (2014). 24463464 10.1038/nsmb.2764 PMC3950333

[b28] Horakova A. H., Moseley S. C., McLaughlin C. R., Tremblay D. C. & Chadwick B. P. The macrosatellite DXZ4 mediates CTCF-dependent long-range intrachromosomal interactions on the human inactive X chromosome. Hum. Mol. Genet. 21, 4367–4377 (2012). 22791747 10.1093/hmg/dds270 PMC3459461

[b29] Chapman A. G., Cotton A. M., Kelsey A. D. & Brown C. J. Differentially methylated CpG island within human XIST mediates alternative P2 transcription and YY1 binding. BMC Genet. 15, 89 (2014). 25200388 10.1186/s12863-014-0089-4 PMC4363909

[b30] Moseley S. C. . YY1 associates with the macrosatellite DXZ4 on the inactive X chromosome and binds with CTCF to a hypomethylated form in some male carcinomas. Nucleic Acids Res. 40, 1596–1608 (2012). 22064860 10.1093/nar/gkr964 PMC3287207

[b31] Cotton A. M. . Landscape of DNA methylation on the X chromosome reflects CpG density, functional chromatin state and X-chromosome inactivation. Hum. Mol. Genet. 24, 1528–1539 (2015). 25381334 10.1093/hmg/ddu564 PMC4381753

[b32] Cotton A. M. . Spread of X-chromosome inactivation into autosomal sequences: role for DNA elements, chromatin features and chromosomal domains. Hum. Mol. Genet. 23, 1211–1223 (2014). 24158853 10.1093/hmg/ddt513 PMC4051349

[b33] Figueroa D. M., Darrow E. M. & Chadwick B. P. Two novel DXZ4-associated long noncoding RNAs show developmental changes in expression coincident with heterochromatin formation at the human (Homo sapiens) macrosatellite repeat. Chromosome Res. 23, 733–752 (2015). 26188586 10.1007/s10577-015-9479-3 PMC4668219

[b34] Darrow E. M. . A region of euchromatin coincides with an extensive tandem repeat on the mouse (*Mus musculus*) inactive X chromosome. Chromosome Res. 22, 335–350 (2014). 24821208 10.1007/s10577-014-9424-x

[b35] McLaughlin C. R. & Chadwick B. P. Characterization of DXZ4 conservation in primates implies important functional roles for CTCF binding, array expression and tandem repeat organization on the X chromosome. Genome Biol. 12, R37 (2011). 21489251 10.1186/gb-2011-12-4-r37 PMC3218863

[b36] Berletch J. B., Yang F., Xu J., Carrel L. & Disteche C. M. Genes that escape from X inactivation. Hum. Genet. 130, 237–245 (2011). 21614513 10.1007/s00439-011-1011-z PMC3136209

[b37] Deng X. . Bipartite structure of the inactive mouse X chromosome. Genome Biol. 16, 152 (2015). 26248554 10.1186/s13059-015-0728-8 PMC4539712

[b38] Chadwick B. P. Macrosatellite epigenetics: the two faces of DXZ4 and D4Z4. Chromosoma 118, 675–681 (2009). 19690880 10.1007/s00412-009-0233-5

[b39] Rao S. S. . A 3D map of the human genome at kilobase resolution reveals principles of chromatin looping. Cell 159, 1665–1680 (2014). 25497547 10.1016/j.cell.2014.11.021 PMC5635824

[b40] Yang F. . The lncRNA Firre anchors the inactive X chromosome to the nucleolus by binding CTCF and maintains H3K27me3 methylation. Genome Biol. 16, 52 (2015). 25887447 10.1186/s13059-015-0618-0 PMC4391730

[b41] Sheffield N. C. . Patterns of regulatory activity across diverse human cell types predict tissue identity, transcription factor binding, and long-range interactions. Genome Res. 24, 1224–1235 (2014). 23482648 10.1101/gr.152140.112 PMC3638134

[b42] Sun L. . Long noncoding RNAs regulate adipogenesis. Proc. Natl Acad. Sci. USA 110, 3387–3392 (2013). 23401553 10.1073/pnas.1222643110 PMC3587215

[b43] Abe Y. . Xq26.1-26.2 gain identified on array comparative genomic hybridization in bilateral periventricular nodular heterotopia with overlying polymicrogyria. Dev. Med. Child. Neurol. 56, 1221–1224 (2014). 25052774 10.1111/dmcn.12553

[b44] Derrien T. . The GENCODE v7 catalog of human long noncoding RNAs: analysis of their gene structure, evolution, and expression. Genome Res. 22, 1775–1789 (2012). 22955988 10.1101/gr.132159.111 PMC3431493

[b45] Cabili M. N. . Integrative annotation of human large intergenic noncoding RNAs reveals global properties and specific subclasses. Genes Dev. 25, 1915–1927 (2011). 21890647 10.1101/gad.17446611 PMC3185964

[b46] Price A. L., Jones N. C. & Pevzner P. A. *De novo* identification of repeat families in large genomes. Bioinformatics 21, i351–i358 (2005). 15961478 10.1093/bioinformatics/bti1018

[b47] Benson G. Tandem repeats finder: a program to analyze DNA sequences. Nucleic Acids Res. 27, 573–580 (1999). 9862982 10.1093/nar/27.2.573 PMC148217

[b48] Katoh K., Misawa K., Kuma K. & Miyata T. MAFFT: a novel method for rapid multiple sequence alignment based on fast Fourier transform. Nucleic Acids Res. 30, 3059–3066 (2002). 12136088 10.1093/nar/gkf436 PMC135756

[b49] Ellison C. E. & Bachtrog D. Non-allelic gene conversion enables rapid evolutionary change at multiple regulatory sites encoded by transposable elements. Elife doi:10.7554/eLife.05899 (2015). 10.7554/eLife.05899 PMC4384637 25688566

[b50] Hoffmann S. . Fast mapping of short sequences with mismatches, insertions and deletions using index structures. PLoS Comput. Biol. 5, e1000502 (2009). 19750212 10.1371/journal.pcbi.1000502 PMC2730575

[b51] Sandelin A., Alkema W., Engstrom P., Wasserman W. W. & Lenhard B. JASPAR: an open-access database for eukaryotic transcription factor binding profiles. Nucleic Acids Res. 32, D91–D94 (2004). 14681366 10.1093/nar/gkh012 PMC308747

[b52] Beckstette M., Homann R., Giegerich R. & Kurtz S. Fast index based algorithms and software for matching position specific scoring matrices. BMC Bioinformatics 7, 389 (2006). 16930469 10.1186/1471-2105-7-389 PMC1635428

[b53] Degner S. C. . CCCTC-binding factor (CTCF) and cohesin influence the genomic architecture of the Igh locus and antisense transcription in pro-B cells. Proc. Natl Acad. Sci. USA 108, 9566–9571 (2011). 21606361 10.1073/pnas.1019391108 PMC3111298

[b54] Parelho V. . Cohesins functionally associate with CTCF on mammalian chromosome arms. Cell 132, 422–433 (2008). 18237772 10.1016/j.cell.2008.01.011

[b55] Ong C. T. & Corces V. G. CTCF: an architectural protein bridging genome topology and function. Nat. Rev. Genet. 15, 234–246 (2014). 24614316 10.1038/nrg3663 PMC4610363

[b56] Gorkin D. U., Leung D. & Ren B. The 3D genome in transcriptional regulation and pluripotency. Cell Stem Cell 14, 762–775 (2014). 24905166 10.1016/j.stem.2014.05.017 PMC4107214

[b57] Rubio E. D. . CTCF physically links cohesin to chromatin. Proc. Natl Acad. Sci. USA 105, 8309–8314 (2008). 18550811 10.1073/pnas.0801273105 PMC2448833

[b58] Cabili M. N. . Localization and abundance analysis of human lncRNAs at single-cell and single-molecule resolution. Genome Biol. 16, 20 (2015). 25630241 10.1186/s13059-015-0586-4 PMC4369099

[b59] Raj A., van den Bogaard P., Rifkin S. A., van Oudenaarden A. & Tyagi S. Imaging individual mRNA molecules using multiple singly labeled probes. Nat. Methods 5, 877–879 (2008). 18806792 10.1038/nmeth.1253 PMC3126653

[b60] Wang J. . Primate-specific endogenous retrovirus-driven transcription defines naive-like stem cells. Nature 516, 405–409 (2014). 25317556 10.1038/nature13804

[b61] Loewer S. . Large intergenic non-coding RNA-RoR modulates reprogramming of human induced pluripotent stem cells. Nature Genet. 42, 1113–1117 (2010). 21057500 10.1038/ng.710 PMC3040650

[b62] Fort A. . Deep transcriptome profiling of mammalian stem cells supports a regulatory role for retrotransposons in pluripotency maintenance. Nature Genet. 46, 558–566 (2014). 24777452 10.1038/ng.2965

[b63] Lu X. . The retrovirus HERVH is a long noncoding RNA required for human embryonic stem cell identity. Nat. Struct. Mol. Biol. 21, 423–425 (2014). 24681886 10.1038/nsmb.2799

[b64] Hasegawa Y. . The matrix protein hnRNPU is required for chromosomal localization of Xist RNA. Dev. Cell 19, 469–476 (2010). 20833368 10.1016/j.devcel.2010.08.006

[b65] Holohan E. E. . CTCF genomic binding sites in Drosophila and the organisation of the bithorax complex. PLoS Genet. 3, e112 (2007). 17616980 10.1371/journal.pgen.0030112 PMC1904468

[b66] Wendt K. S. . Cohesin mediates transcriptional insulation by CCCTC-binding factor. Nature 451, 796–801 (2008). 18235444 10.1038/nature06634

[b67] Reddy T. E. Effects of sequence variation on different allelic transcription factor occupancy and gene expression. Genome Res. 22, 860–869 (2012). 22300769 10.1101/gr.131201.111 PMC3337432

[b68] Horakova A. H. . The mouse DXZ4 homolog retains Ctcf binding and proximity to Pls3 despite substantial organizational differences compared to the primate macrosatellite. Genome Biol. 13, R70 (2012). 22906166 10.1186/gb-2012-13-8-r70 PMC3491370

[b69] Saitou N. & Nei M. The neighbor-joining method: a new method for reconstructing phylogenetic trees. Mol. Biol. Evol. 4, 406–425 (1987). 3447015 10.1093/oxfordjournals.molbev.a040454

