# Supplementary material for: Function and evolution of local repeats in the Firre locus
Source: Nat Commun. 2016 Mar 24;7:11021. doi: 10.1038/ncomms11021 (PMC4820808; doi:10.1038/ncomms11021)
Supplement: Supplementary Information — Supplementary Figures 1-5 and Supplementary Tables 1-4 [file ncomms11021-s1.pdf]

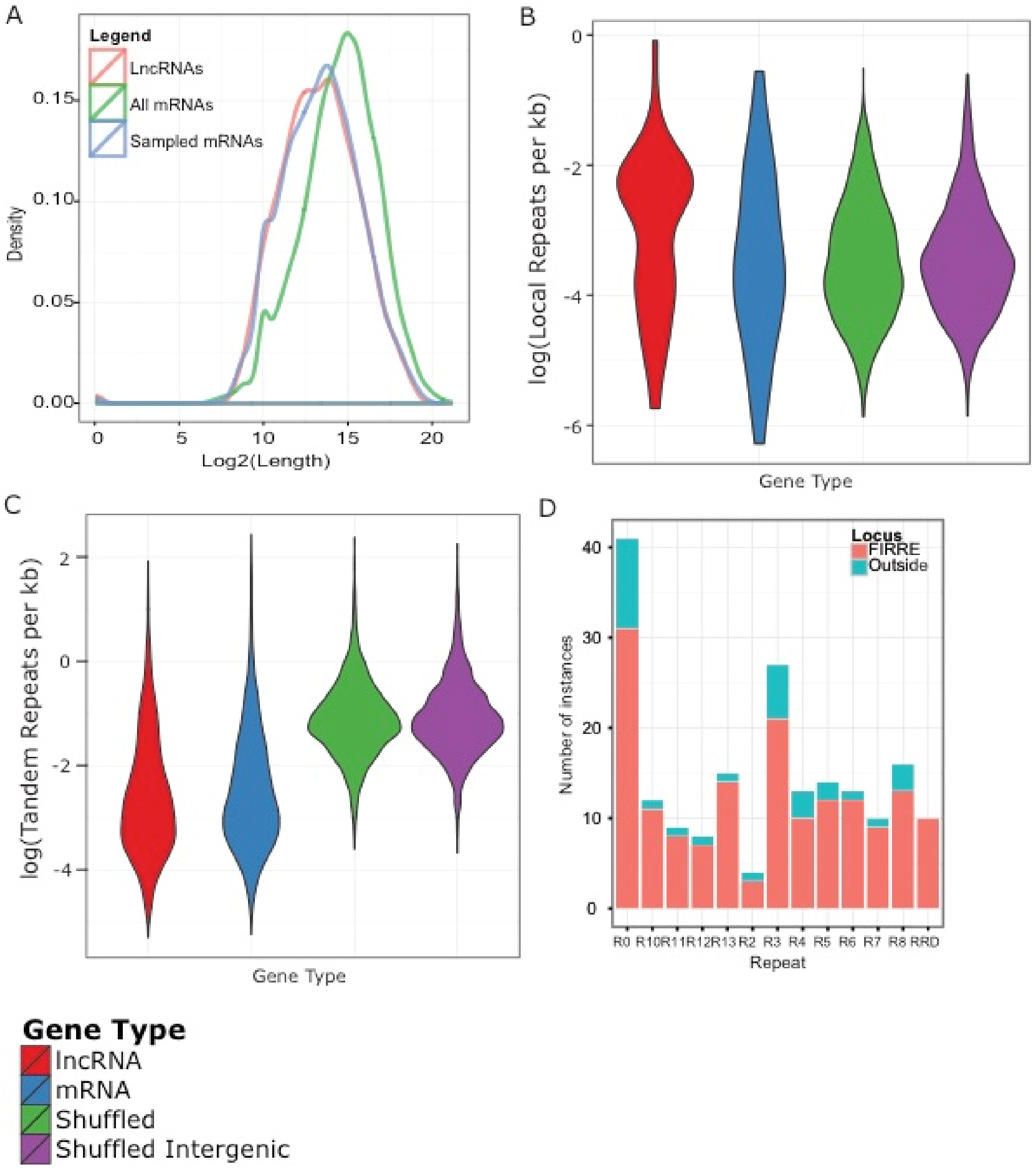

Supplementary Figure 1: The distribution of local and tandem repeats in mRNAs and lncRNAs, including the *FIRRE* locus. (A) Distribution of gene length for mRNAs (green), lncRNAs (red) and the sampled mRNAs (blue) used for this study. (B) Local and tandem repeat distribution for lncRNAs and mRNAs. Violin plot showing local repeats per kb in different gene types and the control sets. (C) Violin plot showing local repeats per kb in different gene types and the control sets. (D) A plot showing the counts for each novel local repeat inside the *FIRRE* locus (red) and outside the *FIRRE* locus (blue).

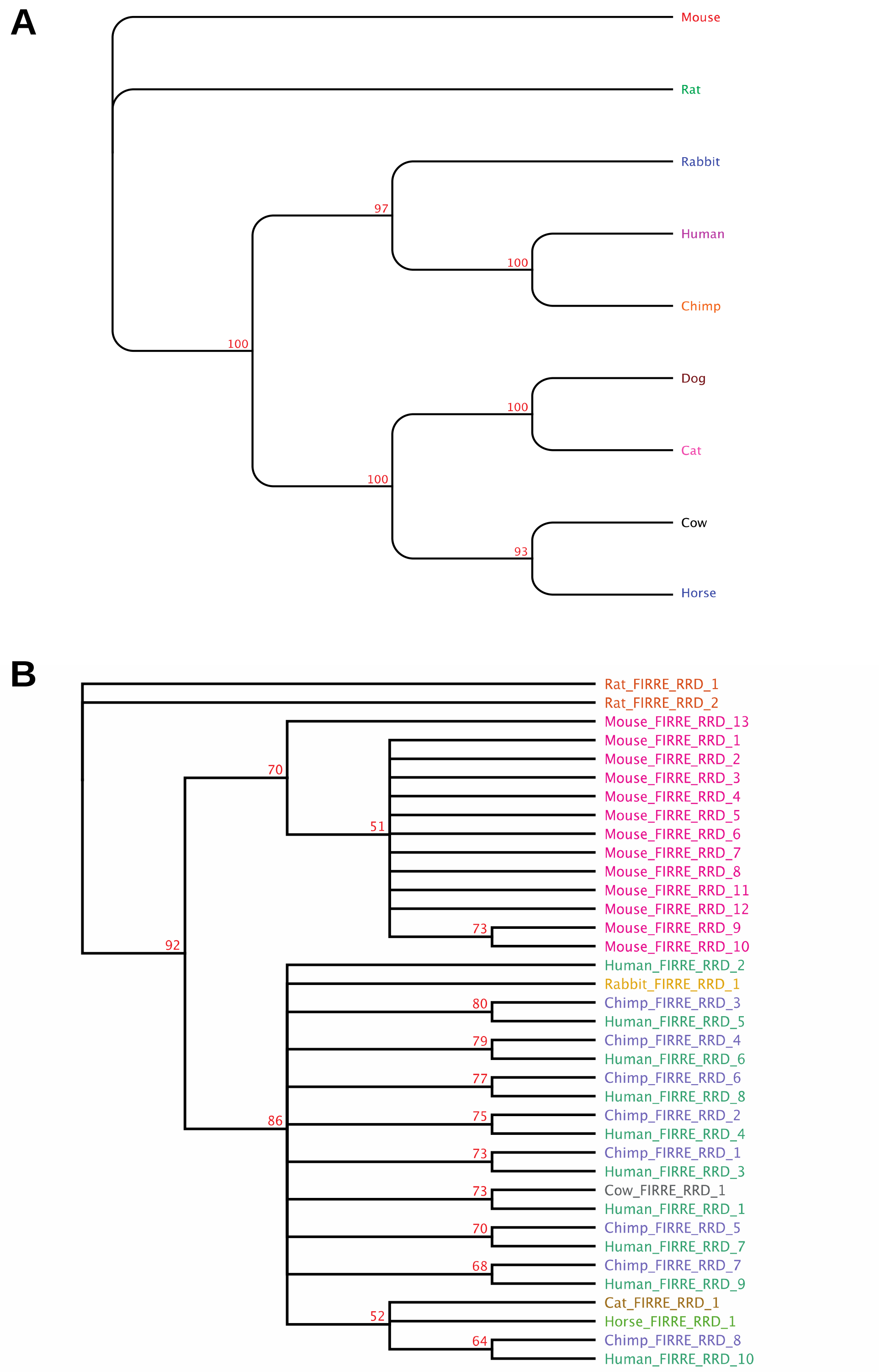

Supplementary Figure 2: **Evolution of the *FIRRE* locus and the repeat RRD.** Using a similar approach as in Figure 3A, we constructed phylogenetic trees of the *FIRRE* locus and RRD from mammals of different orders.

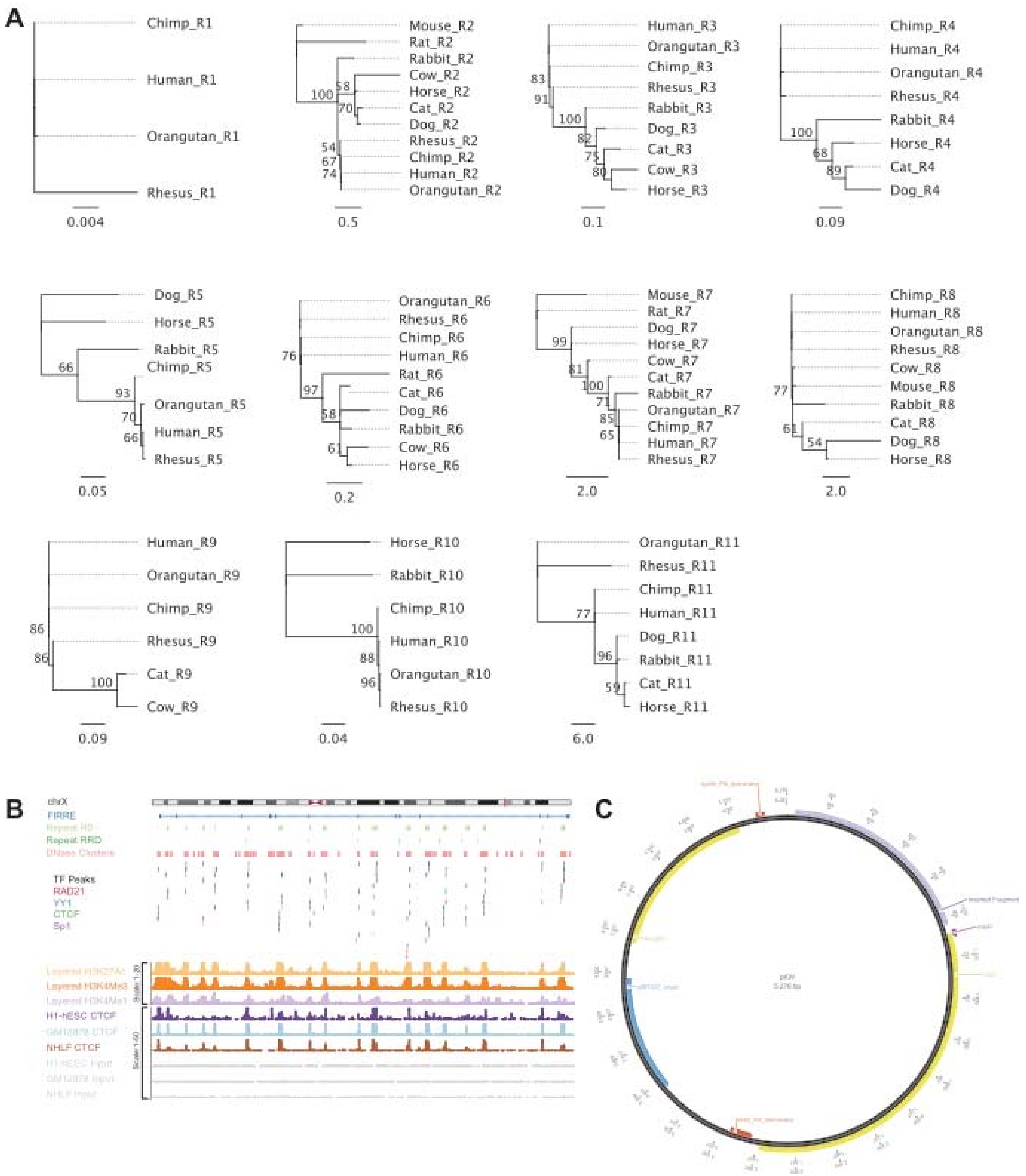

Supplementary Figure 3: Evolution of *FIRRE* LRs and epigenetic marks at repeat R0. (A) Phylogenetic tree of each LR in the *FIRRE* locus**.** (B) UCSC browser screenshot showing the human *FIRRE* locus with the R0 and RRD LRs, DNase clusters, TF peaks

for Sp1, YY1, RAD21, and CTCF in GM12878 and NHLF cell lines. Shown are histone modifications of H3K4Me1, H3K27Ac, H3K4Me3 and CTCF and ChIP-Seq input coverage in hESCs, GM12878 and NHLF cell lines. (C) Generalized vector map of pKW01-pKW14 plasmid for the luciferase enhancer assay. Fragments cloned from the mouse *Firre* locus were inserted as depicted (Inserted Fragment) upstream of a minimal promoter (minP) driving the expression of a Firefly luciferase reporter gene (luc2). The vector also contains an ampicillin resistance selectable marker (amp(R)), a bacterial origin of replication (pBR322_origin), and several transcriptional terminators (SV40_PA_terminator, synth_PA_terminator).

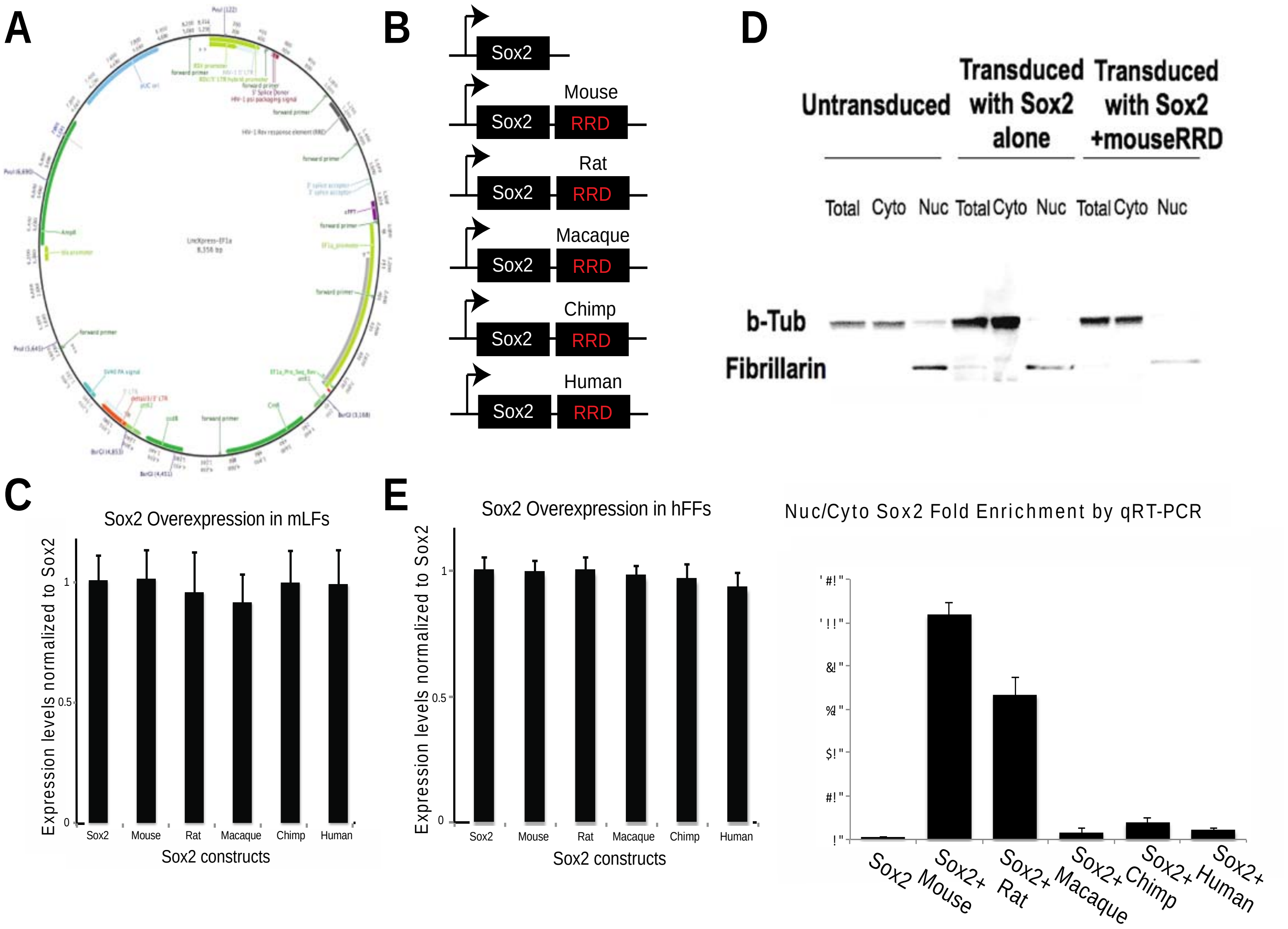

Supplementary Figure 4: **Overexpression of Sox2 constructs.** (A) LincXpress vector map with the modifications to specifically overexpress lncRNAs. (B) The cloned LincXpress Sox2 constructs used for viral transductions. (C) The relative levels of overexpression of Sox2 constructs in mLFs as measured by qRT-PCR. Normalized to the overexpression condition of Sox2 alone. n=3. (D) Biochemical fractionation of mLFs transduced with Sox2 constructs, measured by Western blots to confirm the quality of fractionation and qRT-PCR to measure the transcript levels in nuclear vs cytoplasmic compartments. Normalized to total RNA levels in each compartment. (E) The same analysis as in (C) in hFFs.

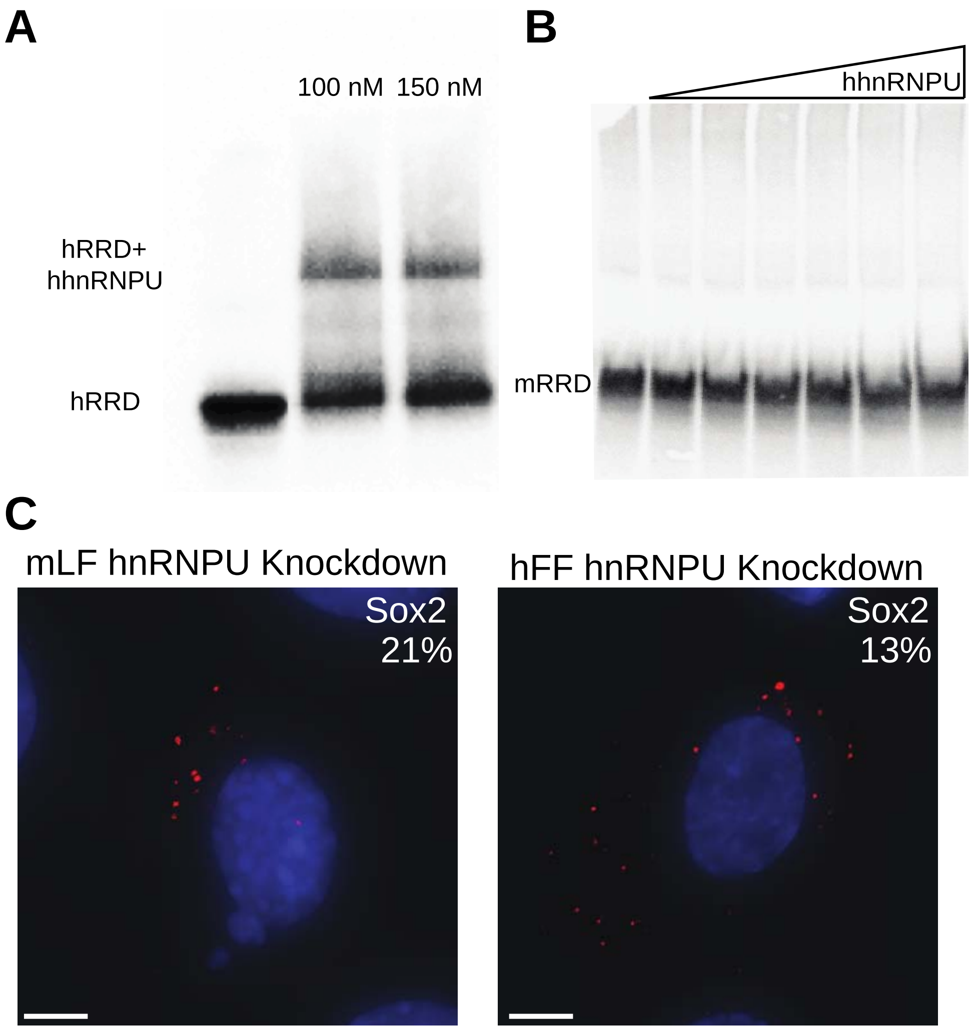

Supplementary Figure 5: High affinity species-specific RRD-hnRNPU interaction and its specific effect on nuclear localization. (A) EMSA using purified human RRD and human hnRNPU. Kd~180 nM. **(B)** EMSA using purified mouse RRD and human hnRNPU Kd is > 1 μM. **(C)** Sox2 without RRD stays cytoplasmic upon hnRNPU knockdown. smRNA FISH using Alexa 594 probes targeting the Sox2 exon. Scale bar: 10 μm for mLF and 15 μm for hFF. Sox2 nuclear localization percentages are included.

Supplementary Table 1: The properties of the local repeats in the *FIRRE* locus

Name of Repeat	Length (bp)	Number of Occurences
R0	701	40
R1	67	3
R2	804	26
R3	229	12
R4	234	13
R5	167	12
R6	255	9
R7	531	15
R8	249	11
R9	78	8
R10	140	7
R11	101	14
RRD	152	10

Supplementary Table 2: The cross-species sequence identity of *FIRRE* local repeats

Repeat	Human-Chimp % Identity	Human-Mouse % Identity
R0	86%	58%
R3	92%	63%
R8	85%	55%
R10	89%	63%
RRD	91%	65%

Supplementary Table 3: The fragments used in the luciferase enhancer assay

Plasmid Name	Primer Fwd	Primer Rev	Genomic Locus (mm9)		
**pKW01**	TTTAAGACAAGGTAAAACCTGCTCC	TCTGGGGCCATACTGCGATT	chr X	47919249	47920241
**pKW02**	AAGAACATGCGCTGTAGCTCT	CTCCTGCTGTCTAATTGGTCACTAT	chr X	47932066	47933638
**pKW03**	GCATGGAGCACAGAGAAGGT	CAGCCTCCTTAGGTTTGGCA	chr X	47964544	47965780
**pKW04**	CACACAAGAGCCCCAGACAT	TGCTGAGCCCTGGGTATAGT	chr X	47920996	47922150
**pKW05**	CCAGCCTGCCTCATGTACTTA	CATCCTGCTTCTCCTCAGCC	chr X	47925181	47926014
**pKW06**	TATATGGAACCTCAGTGTCTGCATT	AGCACAGCAGTGTAAGCTTGT	chr X	47945285	47946258
**pKW07**	GCCCTTTACTCTTCCTTTTCAGC	TCCTACTGCTCCTCAGCCAT	chr X	47954764	47956191
**pKW08**	TGGTAGCATGGTCTGAGATATTTTT	AGCTGCCATAGTTGTTGGACT	chr X	47960576	47961639
**pKW09**	AAAAGAGTCCCACCACCTCG	AGACAAGGGAGGCTAGAGCA	chr X	47967521	47968358
**pKW12**	TAGTTCACTAGGACCACGGC	CTATCTATGAAGCCCTAGTTGCATT	chr X	47969978	47971193
**pKW13**	AACCCAGGCTCTAGGATACGG	ATGGTAGGCTCTGGATGGAGACTT	chr X	47916736	47918348
**pKW14**	CTGGGAGTCCTTTCAATTTCTTTG	CCACGATCCAGTTACACCTCC	chr X	47926970	47928759

Supplementary Table 4: Cloning and qRT-PCR primer sequences

Gateway Cloning Primers

Gene name	Forward
Sox2	GGGGACAAGTTTGTACAAAAAAGCAGGCTTAATG TAC AAC ATG ATG GAG ACG GAG CT
H_HNRNPU	GGG GAC AAG TTT GTA CAA AAA AGC AGG CTT CAT GGA GCT AGG AGA GGA GAA
M-Hnrnpu	GGG GAC AAG TTT GTA CAA AAA AGC AGG CTT CAT GAG TTC TTC GCC TGT TAA
Gene name	Reverse
Sox2	GGGGACCACTTTGTACAAGAAAGCTGGGTATCACATGTGTGAGAGGGGCA
H_HNRNPU	GGG GAC CAC TTT GTA CAA GAA AGC TGG GTC CTA ATA ATA TCC TTG GTG ATA ATG
M-Hnrnpu	GGG GAC CAC TTT GTA CAA GAA AGC TGG GTC CTA ATA ATA TCC TTG GTG ATA ATG

Gibson Cloning Primers

	Forward
Mouse	CAGCTTTCTTGTACAAAGTGGTGGTACGAACTGGCCCCTGGG
Rat	CAGCTTTCTTGTACAAAGTGGTGGTACTAAGTGATCCCAGAGATCGCT
Macaque	CAGCTTTCTTGTACAAAGTGGTGGTACGGCATAGCTCACTAAGGTCTGT
Chimp	CAGCTTTCTTGTACAAAGTGGTGGTACGGC ATG GAT CAC TAA GGT CTG T
Human	CAGCTTTCTTGTACAAAGTGGTGGTACGCATGGAAACCTAAGGTCTGT
**Gibson arms**	**CAGCTTTCTTGTACAAAGTGGTGGTAC**
	Reverse
Mouse	CCCTTCCAGTCCCCGGTACGCTAGCCTTCCTCATTCCC
Rat	CCCTTCCAGTCCCCGGTACTGCCTCATTCCTCTTCTCTTGGGA
Macaque	CCCTTCCAGTCCCCGGTACCCTCATAAAGTATCTCCTCTCTTTAAGAAC
Chimp	CCCTTCCAGTCCCCGGTACCCTCATAAAGTATCTCCTCTCTTTAAGAAC
Human	CCCTTCCAGTCCCCGGTACTGCCCTCATCAAGCATCTCCT
**Gibson arms**	**CCCTTCCAGTCCCCGGTAC**

qPCR Primers

Gene Name	Forward	Reverse
mouse hnRNPU		
#1	TGCCTTTTGACACACCGTAG	CAGCCACCTGTTGAAGAAGA
#2	TGAGGAGACTTGGCTCTGCT	CGTTAAAAGACCGCGAGAAG
human hnRNPU		
#1	GGGGTGTTAAAAGACCACGA	TGAGGAGATTTGGCTCTGCT
#2	ATCTCCTCAGCCACCTGTTG	GGGAAGAAGCACTGAGACGA
mouse Gapdh	GGGAAATTCAACGGCACAGT	AGATGGTGATGGGCTTCCC
human GAPDH	TTCGACAGTCAGCCGCATCTTCTT	GCCCAATACGACCAAATCCGTTGA
